# Microtubule stabilization targeting regenerative chondrocyte cluster for cartilage regeneration

**DOI:** 10.7150/thno.85077

**Published:** 2023-06-12

**Authors:** Jiawei Li, Chunmei Fan, Zhongyang Lv, Ziying Sun, Jie Han, Maochun Wang, Huiming Jiang, Kuoyang Sun, Guihua Tan, Hu Guo, Anlong Liu, Heng Sun, Xingquan Xu, Rui Wu, Wenjin Yan, Qing Jiang, Shiro Ikegawa, Xiao Chen, Dongquan Shi

**Affiliations:** 1 State Key Laboratory of Pharmaceutical Biotechnology, Department of Sports Medicine and Adult Reconstructive Surgery, Affiliated Drum Tower Hospital, Medical School, Nanjing University, 321 Zhongshan Road, Nanjing 210008, Jiangsu, PR China.; 2 Dr. Li Dak Sum-Yip Yio Chin Center for Stem Cells and Regenerative Medicine and Department of Orthopedic Surgery of The Second Affiliated Hospital, Zhejiang University School of Medicine, Hangzhou 310000, Zhejiang, PR China.; 3 Department of Orthopedic Surgery, The Second Affiliated Hospital and Yuying Children's Hospital of Wenzhou Medical University, Wenzhou, 325200, Zhejiang, PR China.; 4 Key Laboratory of Novel Targets and Drug Study for Neural Repair of Zhejiang Province, Department of Clinical Medicine, School of Medicine, Hangzhou City University, Hangzhou, 310000, Zhejiang, PR China.; 5 Key Laboratory of Tissue Engineering and Regenerative Medicine of Zhejiang Province, Zhejiang University School of Medicine, Hangzhou 310000, Zhejiang, PR China.; 6 Department of Sports Medicine and Adult Reconstructive Surgery, the Affiliated Nanjing Hospital of Nanjing Medical University, Nanjing 210000, Jiangsu, PR China.; 7 Laboratory for Bone and Joint Diseases, RIKEN Center for Integrative Medical Science (IMS, RIKEN), Tokyo 108-8639, Japan.; 8 Department of Sports Medicine, Zhejiang University School of Medicine, Hangzhou, PR China.; 9 China Orthopedic Regenerative Medicine Group (CORMed), Hangzhou 310000, Zhejiang, PR China.

**Keywords:** Cartilage regeneration, Chondrocytes, Microtubule stabilization, Single cell RNA sequencing, Yes associated protein

## Abstract

**Purpose:** Chondrocytes (CHs) in cartilage undergo several detrimental events during the development of osteoarthritis (OA). However, the mechanism underlying CHs regeneration involved in pathogenesis is largely unknown. The aim of this study was to explore the underlying mechanism of regeneration of CHs involved in the pathological condition and the potential therapeutic strategies of cartilage repair.

**Methods and Materials:** CHs were isolated from human cartilage in different OA stages and the high-resolution cellular architecture of human osteoarthritis was examined by applying single-cell RNA sequencing. The analysis of gene differential expression and gene set enrichment was utilized to reveal the relationship of cartilage regeneration and microtubule stabilization. Microtubule destabilizer (nocodazole) and microtubule stabilizer (docetaxel) treated-human primary CHs and rats cartilage defect model were used to investing the effects and downstream signaling pathway of microtubule stabilization on cartilage regeneration.

**Results:** CHs subpopulations were identified on the basis of their gene markers and the data indicated an imbalance caused by an increase in the degeneration and disruption of CHs regeneration in OA samples. Interestingly, the CHs subpopulation namely CHI3L1^+^ CHs, was characterized by the cell regenerative capacity, stem cell potency and the activated microtubule (MT) process. Furthermore, the data indicated that MT stabilization was effective in promoting cartilage regeneration in rats with cartilage injury model by inhibiting YAP activity.

**Conclusion:** These findings lead to a new understanding of CHs regeneration in the OA pathophysiology context and suggest that MT stabilization is a promising therapeutic target for OA and cartilage injury.

## Introduction

Articular cartilage is an organized tissue that allows smooth motion and load-bearing in mammalian synovial joints 1. Osteoarthritis (OA) is the most common disorder that contributes to the degeneration of cartilage 2. During OA progression, cartilage resident chondrocytes (CHs) undergo several pathological events, such as inflammation, mechanical stimulation, oxidative stress, and apoptosis 1. Unfortunately, the hypocellularity and avascular features of cartilage coupled with the inability of resident CHs or progenitor cells to generate a hyaline extracellular matrix (ECM) lead to the unsatisfactory result after current therapeutic strategies which are applied to cartilage regeneration 3. In recent decades, much attentions have been directed to the exploration of signaling mechanism underlying cartilage degeneration in OA, but little attention has been given to their regeneration. A lack of knowledge about the basic biology, formation and maintenance of the hyaline cartilage phenotype, and the molecular mechanism of CHs regeneration in the pathological condition of OA are important reason for the failure of cartilage regeneration in basic research and clinical settings.

Although, the CHs have been identified as the single cell type found in cartilage tissue, the underlying molecular mechanism of CHs action is complicated due to the heterogeneity of cellular function and the complex microenvironment 4. Recently, the transcriptional program in OA contexts and various types of CHs have been identified during OA development by single cell RNA sequence. The relationship between these different CHs subtypes has aided in the identification of the explicit mechanism of OA pathogenesis. Understand of specific degeneration-related phenotype of each CHs subpopulation and the crosstalk among cells and molecules has increased the number of OA therapeutic targets identified to date 4, 5. These studies focused on degeneration with the aim of identifying the mechanism of OA development as the presumed basis for OA progression prevention. However, for the issues about the existing cartilage injury and the approach of cartilage repair, more attention needs to be paid to the endogenous capability of cartilage regeneration. The knowledge of CHs regeneration and underlying molecular mechanism during OA development is essential for increasing the diagnostic and therapeutic options and effectiveness for cartilage repair.

A balance between intracellular forces and the cytoskeleton plays key roles in the regulation of basic cellular functions and signal transduction, such as cell proliferation, apoptosis, differentiation, adhesion, and migration 6. Cytoskeleton consists of actin, microtubules (MTs) and intermediate filaments. The actin microfilaments affect the CHs phenotype by regulating cell shape. CHs flattening due to external factors and stress fibers formation in cells trigger dedifferentiation 7. This process is manifested mainly as a decrease in the synthesis of type II collagen (COL II) and Aggrecan, as well as an increase in type I collagen (COL I) synthesis 8, indicating the acquisition of a fibrotic phenotype. MTs are the mainstays for 'sensing' mechanical signals between the cell components and the ECM 9, 10. The stability of MTs ensure the long MT life and the MT extension to guarantee their basic functionality 11. Defects in MT motors and other associated proteins underlie diseases such as Alzheimer's disease, cancer and genetic diseases 12. Over the past few decades, considerable knowledge has been gained with respect to MT structure and function, making these MTs potential therapeutic targets. Many studies have reported that MT stabilization can be leveraged in an effective strategy for disease treatment and cell differentiation regulation 13, 14.

In the present study, the degree of spontaneous regeneration of injured cartilage was determined by single cell RNA sequencing. Interestingly, a CHs subpopulation expressing the biomarker *CHI3L1* was identified and showed the endogenous capability for cartilage repair and stem cell potency. The transcriptome profiling results showed that the cytoskeleton process was enriched in CHI3L1^+^ CHs cluster, especially MT. The current study further demonstrates the role of MT stabilization as an important mechanism involved in articular cartilage restoration.

## Results

### Single-cell atlas of the regeneration and degeneration of human articular chondrocytes

**Figure 1:**
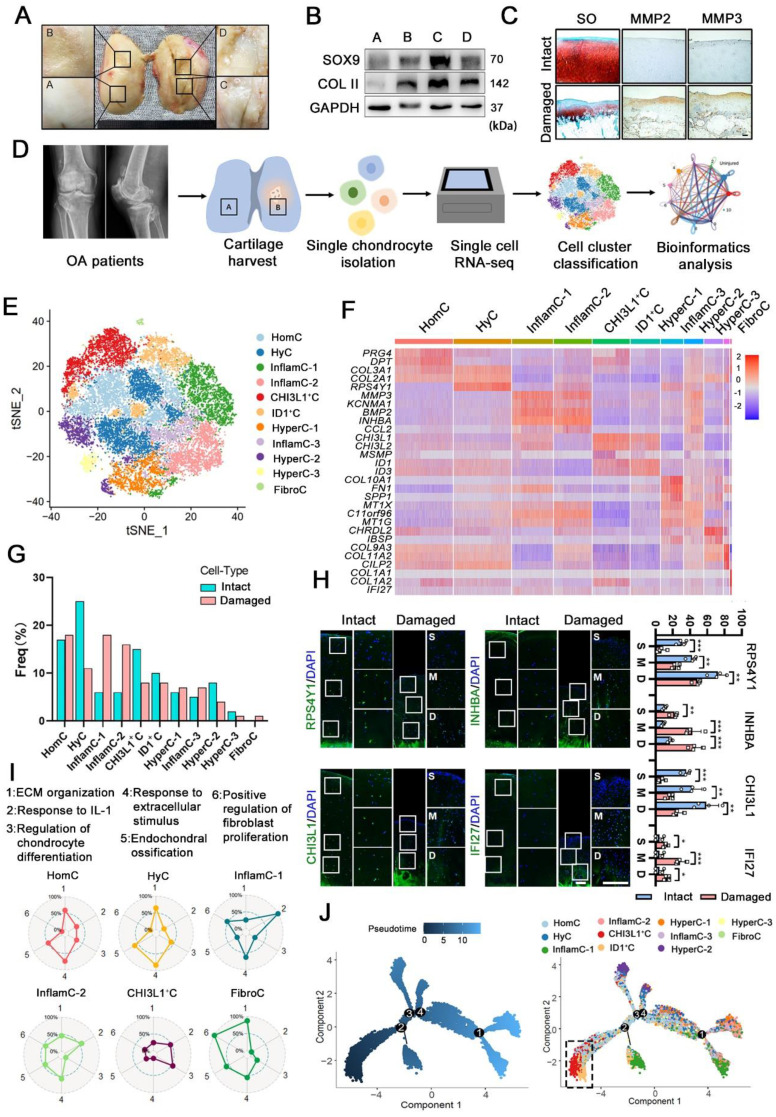
**Single-cell atlas of human articular chondrocyte. (A)** Appearance of a cartilage specimen harvested from a knee OA donor. Four areas of the femoral condyle (A and B: the lateral condyle, C and D: medial condyle) were analysed. **(B)** Western blot analysis of SOX9, and collagen II (COL II) in the four areas of human OA cartilage tissue harvested from the sample shown in A. **(C)** Safranin O and fast green (SO) staining and immunohistochemical staining of matrix metalloproteinase 2 (MMP2) and MMP3 in intact and damaged cartilage. Scale bar, 200 μm. **(D)** Schematic workflow of the experimental strategy.** (E)** t-distributed stochastic neighbour embedding (t-SNE) plot showing the unbiased clustering results of all the filtered cells, cells were coloured by the cluster (Left) and cell type (Right). **(F)** Heatmap showing the relative expression levels of Differentially expressed genes (DEGs) for clusters. **(G)** Fractions of each cell cluster in intact (n = 3) and damaged (n = 3) articular cartilage. **(H)** Representative immunofluorescence staining of markers of the CHs population whose cell composition significantly altered between two groups (HyC, InflamC, CHI3L1^+^C, and FibroC). Enclosed areas are enlarged in right panels. Scale bar, 100 μm. Quantification of immunofluorescence positive cell in different zone [superficial (S), middle (M) and deep (D)]. n = 4.** (I)** Radar map showing the performance of six gene sets associated with the indicated function among each CHs [HomC, HyC, InflamC-1, InflamC-2, CHI3L1^+^C, and FibroC].** (J)** Monocle pseudotime trajectory revealing the OA chondrocyte lineage progression. Data are represented as the mean ± SD. *P < 0.01, **P < 0.01, ***P < 0.001.

First, we investigated the regeneration and degeneration of femoral condyle cartilage in different stages of OA patients undergoing total knee arthroplasty (TKA). Macroscopic and histological investigation indicated that cartilage abrasions in the medial femoral condyle (C&D) was more severe than that in the lateral (A&B) (Figure 1A, 1C). However, it was observed that the damaged cartilage (C&D) exhibited high expression of cartilage regenerative proteins, for example, SOX9 and collagen II (COL II) (Figure 1B). This observation indicated that cartilage may be regenerated via a self-compensatory mechanism under detrimental conditions.

To define key molecular mechanisms involved in cartilage regeneration in OA, human CHs were isolated from the femoral condyles cartilage of 3 patients undergoing TKA (Figure 1D and S1). Then single-cell RNA sequencing was performed (Figure 1D). Following a stringent quality control procedure, we maintained 20,839 cells for use in a downstream analysis and the t-distributed stochastic neighbour embedding (t-SNE) projection revealed 11 distinct cell clusters (Figure 1E and S2A). The markers were identified across all clusters (Figure 1F, S2B and S2C). Based on the expression of these markers and lineage-related genes, we annotated them as follows: homeostasis CHs (HomC), hyaline CHs (HyC), hypertrophic CHs (HyperC-1-3), inflammatory CHs (InflamC-1-3), fibrocartilage CHs (FibroC), *CHI3L1^+^* CHs (CHI3L1^+^C) and *ID1*^+^ CHs (ID1^+^C). Quantification of the cellular composition was confirmed by the expansion of *MMP3^+^INHBA^+^* CHs (InflamC-1&2) as well as a decline in HyC and CHI3L1*^+^*C subpopulation proportions in the damaged cartilage (Figure 1G). The FibroC cluster was the only cell set in the damaged cartilage that the exclusively expressed *COL1A1* and *IFI27* at high levels (Figure 1F and 1G). As shown in Figure 1H, immunofluorescence staining indicated that *RPS4Y1^+^*HyC and *CHI3L1^+^* C were distributed in all zones of intact cartilage, while they were limited in the middle (M) and deep (D) zones of the damaged cartilage. Moreover, a limited number of *INHBA*^+^ InflamC (InflamC-1&2) was located in the deep zone of intact cartilage, and they were extensively expanded to all zones of damaged cartilage. The *IFI27*^+^FibroC was observed largely in the middle zone of the damaged cartilage.

The striking alteration in the cellular composition of these clusters indicated their important role in regulating OA progression. The cellular functions of clusters were assessed via gene set analysis, and the results indicated that the term of ECM organization and the response to the extracellular stimuli played a main role in the metabolic pattern of HomC and HyC (Figure 1I). In addition, both InflamC-1&2 exhibited strong enrichment in the response to interleukin-1 (IL-1). FibroC only appeared in the damaged cartilage and was enriched in ECM organization and positive regulation of fibroblast proliferation. Interestingly, the subpopulation of CHI3L1*^+^*C indicated a relative advantage in the regulation of CHs differentiation. The function evaluated by the gene set of the remaining clusters was also evident by the radar map (Figure S3A). It was found that CHI3L1*^+^*C expressed relatively high levels of stem cell-related genes. Specifically, the following geneswere highly expressed: *ENG (CD105), BIRC5, MYLK, LY6E, THY (CD90), STAG1, ITGB1 (CD29), ALCAM (CD166)* and *VCAM1 (CD106)* (Figure S3B). Ordering of cells in pseudo-time arranged most of CHs into a major trajectory, with three minor bifurcations (Figure 1J). The CHI3L1^+^C was distributed at the start of the trajectory (Figure 1J). The transcriptomic differences between these CHs were compared with those of the previously analyzed CHs at different stages of OA (stages 0-4) 4. The results indicated a correlation and difference between these two datasets (Figure S2D). An analysis of the contribution of each patient's data to the subpopulation was relatively consistent with the proportion of each cell cluster (Figure 1G and S3B). In summary, the single-cell RNA-seq-based transcriptome analysis revealed a significant imbalance in the subpopulations of CHs in damaged cartilage. Furthermore, the functional analysis showed that the CHI3L1^+^C may possess CH regeneration functions and significant stem cell potency in OA cartilage.

### Unique networks of chondrocytes subpopulations in damaged cartilage

**Figure 2:**
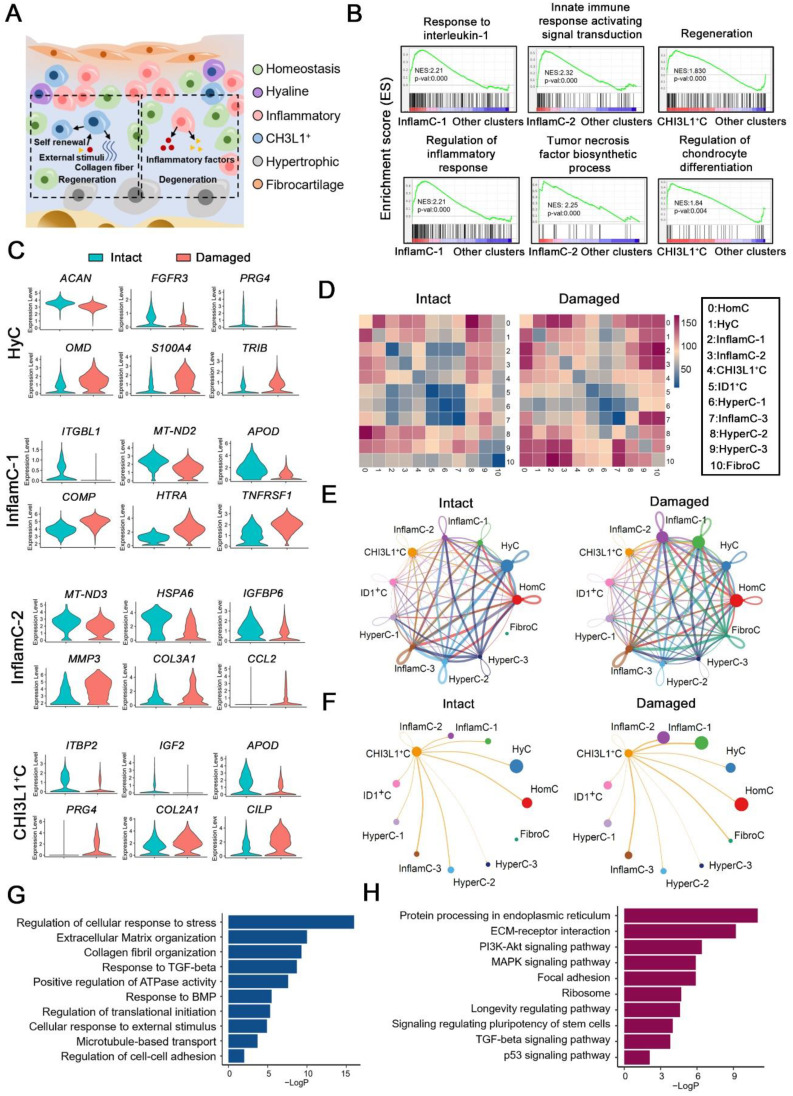
** Multicellular interactions in the damaged chondrocyte niche. (A)** Schematic of six chondrocytes (CHs) subpopulation involved in degeneration and regeneration in OA cartilage. **(B)** GSEA showing enrichment of pathways between InflamC-1, InflamC-2, and CHI3L1^+^C with other clusters, respectively. **(C)** Violin plots showing expression levels of the represented marker genes of intact and damaged CHs of HyC, InflamC-1, InflamC-2 and CHI3L1^+^C. **(D)** Heatmap displaying the numbers of the enriched annotated ligand and receptor pairs between intact and damaged CHs clusters. The CHs clusters represented by number were listed at the right panel. **(E)** Network showing the interaction strength between the cell clusters of the intact and damaged CHs. **(F)** Network showing the interaction strength from CHI3L1^+^C to other cell clusters of the intact and damaged CHs.** (G)** Bar plots showing the enriched Gene Ontology (GO) terms of damaged CHI3L1^+^C. **(H)** Bar plots showing the enriched Kyoto Encyclopaedia of Genes and Genomes (KEGG) terms of damaged CHI3L1^+^C.

OA is characterized by remodeling of the cellular niche 3, which depends on complex interactions between multiple cell subpopulations (Figure 2A). A gene set enrichment analysis (GSEA) indicated that the items that responded to IL-1 and the regulation of the inflammatory response were enriched in InflamC-1. The innate immune response activating signaling transduction and tumor necrosis factor biosynthetic process were enriched in InflamC-2 (Figure 2B). In addition, CHI3L1^+^C was primarily engaged in regeneration and CH differentiation (Figure 2B). Subsequently, we mainly focused on the differentially expressed genes between damaged and intact CHs. In HyC, the levels of *ACAN*, *FGFR3* and *PRG4* declined, while those of *OMD, S100A4* and *TRIB* were enhanced in the damaged cartilage (Figure 2C). The decrease of *ITGBL1*, *MT-ND2* and *APOD* as well as the increase of *COMP*, *HTRA* and *TNFRSF12A* were demonstrated in pathological InflamC-1 (Figure 2C). The pathological CHs of InflamC-2 were shown to express *MMP3, COL3A1* and *CCL2* and demonstrated weak expression of *MT-ND3, HSPA6* and *IGFBP6* (Figure 2C). Importantly, higher expression of CHs regenerative-related genes, such as *PRG4, COL2A1* and *CILP* was observed in the damaged group of CHI3L1^+^C, whereas in the intact group, the expression levels of the homeostasis related genes *IGF2, LTBP2* and *APOD* were elevated (Figure 2C).

To identify the key regulators in the cartilage niche, an unbiased ligand-receptor interaction analysis was performed and the results were compared among CH cell clusters 15. In intact controls, the cell-cell interaction landscape was dominated by the HomC, HyC, HyperC-2 and HyperC-3 (Figure 2D and 2E). In contrast, the cellular communication in the damaged cartilage was changed in the FibroC, InflamC-1 and InflamC-2 (Figure 2D and 2E). The interactions of CHI3L1^+^C with InflamC-1&2 and FibroC were also enhanced in the damaged cartilage (Figure 2F).

A Gene Ontology (GO) analysis revealed that positive regulation of cell death and negative regulation of growth were promoted in the HyC of the damaged cartilage (Figure S4A). The InflamC-1&2 in the damaged cartilage were both enriched in the IL-18 signaling pathway, while they were impaired with regard to the response to oxidative stress and mechanical stimulus (Figure S4B and S4C). The upregulated genes in the pathological CHI3L1^+^C were focused on the GO terms including regulation of cellular response to stress, extracellular matrix organization and response to transforming growth factor (TGF)-beta (Figure 2G and S4D), and the Kyoto Encyclopedia of Genes and Genomes (KEGG) terms including ECM-receptor integration, focal adhesion, the signaling pathways responsible for regulating pluripotency of stem cells and TGF-beta signaling pathway (Figure 2H and S4D). Taken together, these data revealed the specific mechanism by which HyC, InflamC and CHI3L1^+^C interacted in the damaged cartilage. Importantly, the findings further demonstrated that CHI3L1^+^C may be responsible for the regeneration process in OA.

### The relationship of the regenerative potency of CHs with microtubule process

**Figure 3:**
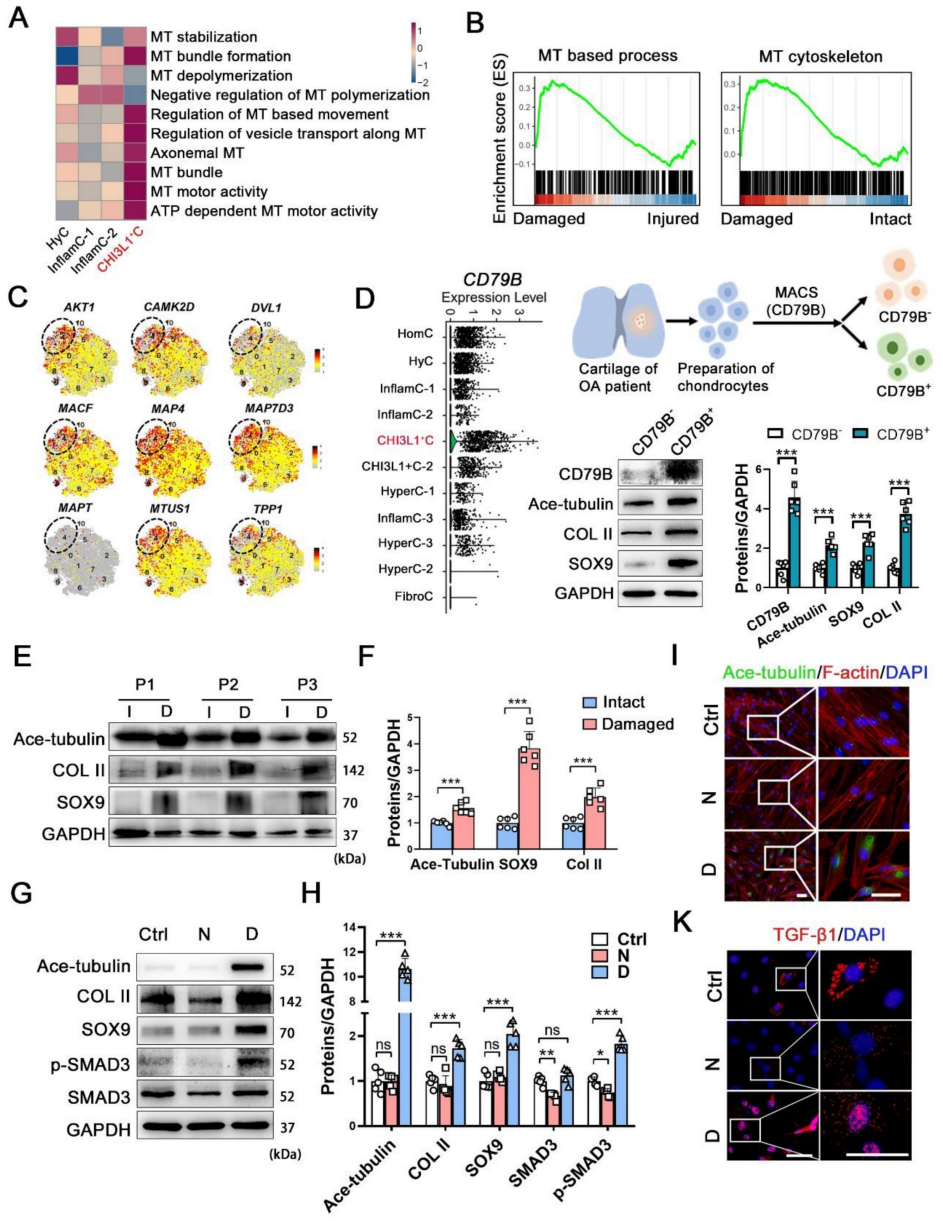
** The relationship among Increased microtubule-related phenotype and CHI3L1^+^C. (A)** GSVA analysis revealing multiple microtubule (MT) process involved in CHI3L1^+^C. **(B)** GSEA analysis revealing several MT-related GO terms positively correlated with damaged CHI3L1^+^C. **(C)** Feature plots showing the relative expression of indicated markers for each cell cluster on the t-SNE map. The CHI3L1^+^C was circled in the white dot line. **(D)** Violin plots showing the expression of *CD79B* in each clusters (left). Schematic of magnetic-activated cell sorting (MACS) of primary chondrocytes (CHs) (upper). Western blot analysis of CD79B, Ace-tubulin, COL II, and SOX9 in CD79B^-^ and CD79B^+^ CHs (lower). **(E)** Western blot analysis of Ace-tubulin, SOX9 and Col II in human cartilage harvested form intact (I) and damaged (D) areas of 3 patients (P1-3). **(F)** Quantification of data E. n = 6. **(G)** Western blot analysis of Ace-tubulin, COL II, SOX9, SMAD3, and phosphorylated SMAD3 expression in CHs treated by nocodazole (N) and docetaxel (D), respectively. **(H)** Quantification of the data of G, n = 5. **(I-K)** Immunofluorescence staining of Ace-tubulin and F-actin (I), and TGF-β1 (K) in CHs treated by nocodazole (N) and docetaxel (D), respectively. Enclosed areas are enlarged in right panels. Scale bar, 50 μm. Increase of MT stability by the docetaxel treatment increased expression levels of COL II, SOX9 and activated SMAD3 signal in CHs. Data are represented as the mean ± SD. *P < 0.05, ***P < 0.001.

To reveal the underlying mechanism related to the regenerative potency of the CHI3L1^+^C, we further explored the features of this cluster. We examined the level of molecules related to cytoskeletal composition and signals (Figure S5A). The Microtubule (MT) and actin-related genes were more highly expressed in CHI3L1^+^C. The genes involved in primary ciliary functions (*IFT88* and *IFT80*) and Hippo signaling pathway (*MST1, LATS1* and *YAP1*) also showed relatively high expression level in CHI3L1^+^C (Figure S5A). Moreover, a gene set variation analysis (GSVA) demonstrated that multiple MT process was involved in CHI3L1^+^C (Figure 3A). The pathological CHI3L1^+^C were highly enriched in MT-based process and MT-cytoskeleton (Figure 3B), revealing that MT process was mainly activated in the CHI3L1^+^C of damaged cartilage. The CHI3L1^+^C was validated by examining the MT stabilization related signals (*AKT1, CAMK2D, DVL1*) and the MT-associated proteins (*MACF, MAP4, MAP7D3, MAPT, MTUS1, TPP1*) (Figure 3C). The data indicated that the CHI3L1^+^C was highly related to MT stabilization. To identify the regeneration and MT-related characteristics of these CHs, the cells in the CHI3L1^+^C cluster were separated by magnetic-activated cell sorting (MACS) using an anti-CD79B antibody, as this protein was found to be specifically enriched in the CHI3L1^+^C (Figure 3D and S5B). Acetylated-α-tubulin (Ace-tubulin) represents stabilized MT 16. This protein is found in long-lived, stable MTs with a low turnover rate 17. The level of Ace-tubulin, COL II and SOX9 were significantly higher in CD79B^+^ CHs than those noted in CD79B^-^ CHs (Figure 3D).

We also investigated the relationship between OA and cytoskeleton by examining another public dataset 18. The levels of the tubulin gene expression were increased in OA cartilage (Figure S6A). The genes of ECM organization, extracellular structure organization and MT polymerization were enriched in OA (Figure S6B). The human OA cartilage samples were classified into an intact and a damaged group, and the positive relationship between MT stability was verified on the basis of SOX9 and COL II expression (Figure 3E-F, and S6C). To determine the relationship between MT stabilization and cartilage regeneration, nocodazole and docetaxel were utilized to destabilize and stabilize MTs for 2 weeks 19, 20, respectively. The Ace-tubulin levels in CHs were significantly increased following treatment with docetaxel for 2 weeks (Figure 3G-I). The results of a cell viability assay indicated that nocodazole enhanced the proliferation rate of CHs when the concentration was higher than 25 nM, while docetaxel did not influence cell viability when administered at 2.5 nM (Figure S6D). A RT-qPCR analysis of CHs treated with different concentrations of nocodazole (5, 10 and 25 nM) and docetaxel (0.5, 1 and 2.5 nM) revealed that MT stabilization led to increased *SOX9* and *COL2A1* levels and decreased *RUNX2* and *COL1A1* levels (Figure S6E). Based on these finding, the doses of 25 nM nocodazole and 2.5 nM docetaxel were selected to treat CHs. The protein levels of COL II, SOX9 and TGF-β1 were increased in the docetaxel group (Figure 3G-H). SMAD3, which is an intracellular mediator of TGF-β signaling, was also activated (Figure 3G-I). We also demonstrated the effects of MT stabilization in CHs with another MT stabilizer, epothilone (Figure S6F). These results identified a regulatory effect of MT stabilization on the maintenance of the function of the CHs.

### Microtubule stabilization enhances cartilage regeneration

**Figure 4:**
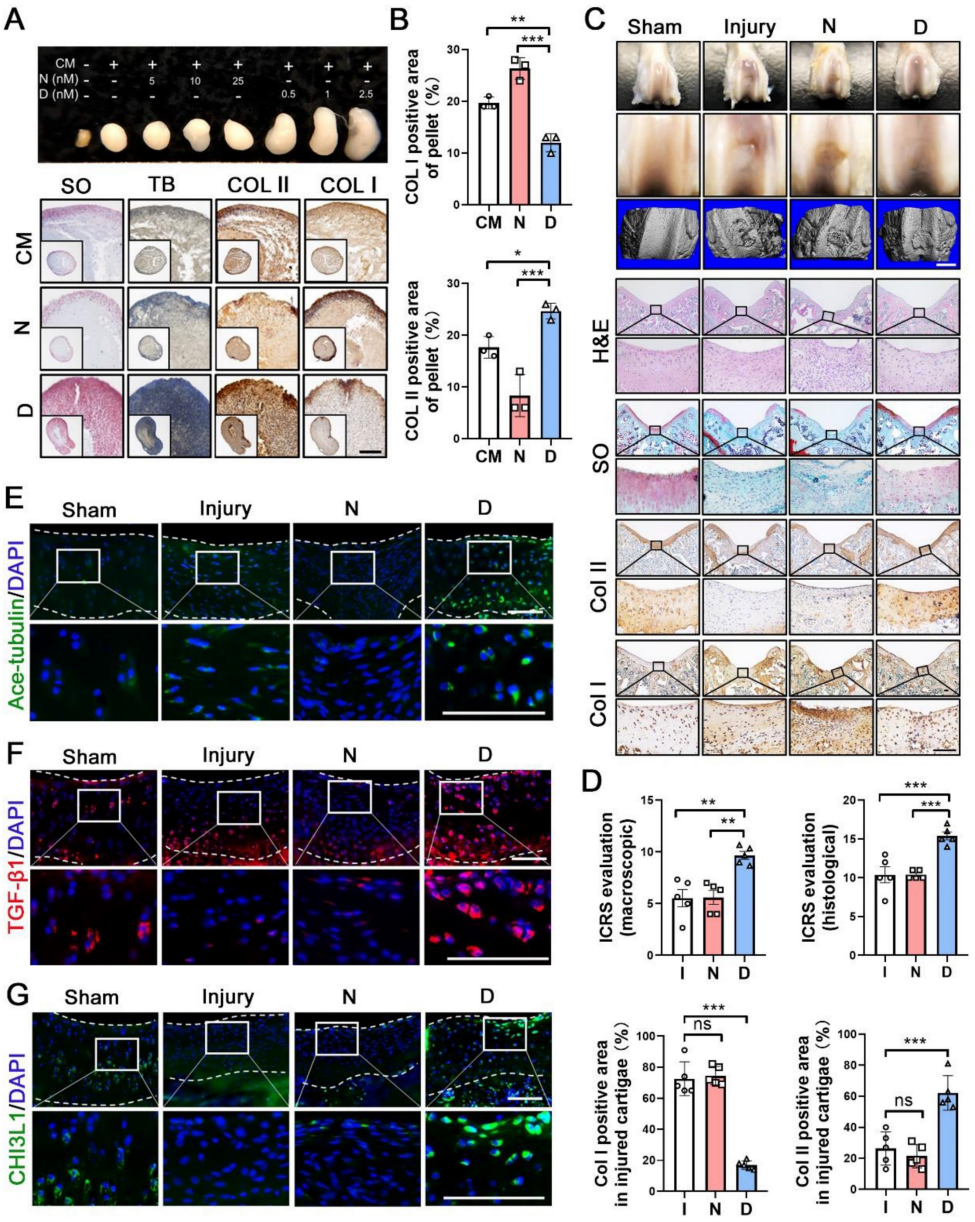
** Microtubule stabilization promotes chondrogenesis and regeneration in human chondrocytes and rat cartilage repair. (A)** Pellet cultures in chondrogenic medium (CM) treated with different concentrations of nocodazole (N) and docetaxel (D) for 4 weeks. SO and toluidine blue (TB) staining and immunohistochemical staining of Col II and Col I of pellets. Scale bar, 100 μm. **(B)** Quantification of data A, n = 3. MT stabilization by docetaxel treatment promoted the chondrogenesis. Scale bar, 200 μm. **(C)** Macroscopic appearance and histology of the cartilage defect in rat knee joint. Top: gross view of femoral condyle, middle: close-up view of the trochlear region, lower: micro-CT 3D reconstruction. White scale bar, 2 mm. H&E, SO staining and immunohistochemical staining of Col II and Col I. Enclosed areas are enlarged in below panels. Black scale bar, 100 μm. **(D)** Quantification of macroscopic and histological ICRS evaluation, and the positive area of Col I and Col II in injured cartilage for the rat injury model in each group [injury (I), nocodazole (N) and docetaxel (D)] for 6 weeks after surgery (n = 5). **(E-G)** Immunofluorescence staining for Ace-tubulin (E), TGF-β1 (F), and CHI3L1 (G) in cartilage injury model. Enclosed areas are enlarged in below panels. Scale bar, 100 μm. Intra-articular injection of docetaxel improved cartilage repair in the injury model. The borders of cartilage with articular cavity and subchondral bone were marked with white dot line. Data are represented as the mean ± SD. *P < 0.05, **P < 0.01, ***P < 0.001.

Having shown the effects of MT stabilization on CHs function, we further examined the role of this mechanism in cartilage repair. Following 28 days of treatment, the pellets treated with docetaxel in the chondrogenic medium (CM) exhibited a smooth and packed structure and were larger than those of the CM control and nocodazole groups (Figure 4A). The histological analysis revealed that a more cartilage-like ECM was formed in the pellet treated with docetaxel (Figure 4A-B). Furthermore, we established a full-thickness defect (2.0 mm in diameter, 3.0 mm in depth) in the center of the trochlear groove in the right knee of the rats and subsequently performed intra-articular injection of nocodazole (50 μg/kg), docetaxel (2 μg/kg) and saline weekly. Following 6 weeks, H&E staining of key organs confirmed the safety from these two reagents (Figure S7A). In the docetaxel group, the defect was markedly filled to a greater extent and with a smoother surface compared to that in the other groups (Figure 4C). Moreover, an analysis of the 3D reconstruction based on micro-CT revealed greater integration of subchondral bone formation in the docetaxel group (Figure 4C). The high expression of Col II and a lack of Col I revealed a hyaline-like ECM in the repaired tissue of the docetaxel group (Figure 4C, 4D and Figure S8). Quantifications of international cartilage regeneration and joint preservation society (ICRS) scores from the macroscopic and histological evaluation indicated that the docetaxel group exhibited significant improvements (Figure 4D). Increased Ace-tubulin expression was noted in the repaired cartilage of the docetaxel treatment group suggesting the significant MT stabilization effect (Figure 4E and S9A). In addition, the expression levels of TGF-β1 and CHI3L1 were markedly higher in the docetaxel-treated cartilage than in the injured and the nocodazole groups (Figure 4F-G and S9A). These findings suggest that MT stabilization in CHs was increased via the intra-articular injection of docetaxel, which led to a significant enhancement of the cartilage regeneration.

### Microtubule stabilization inhibits YAP activity in CHs

**Figure 5:**
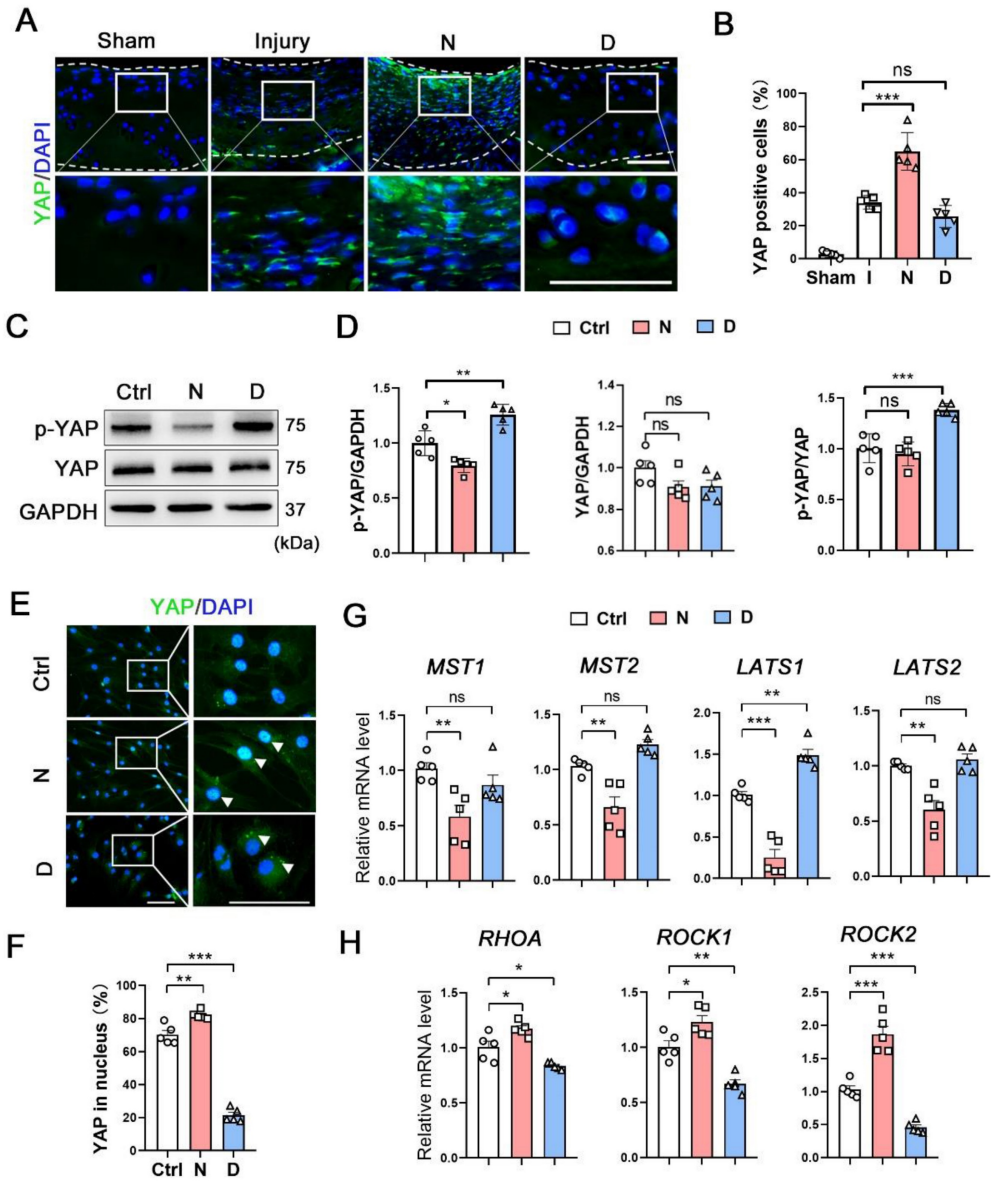
** The role of microtubule stabilization for YAP inhibition. (A)** Immunofluorescence staining for YAP in rat cartilage injury model. Enclosed areas are enlarged in below panels. Scale bar, 100 μm. The borders of cartilage with articular cavity and subchondral bone were marked with white dot line.** (B)** Quantification of data A in each group [injury (I), nocodazole (N) and docetaxel (D)], n = 5. **(C)** Western blot analysis of phosphorylated YAP and YAP treated with nocodazole (N) or docetaxel (D) for 1 week in chondrocytes (CHs). **(D)** Quantification of the data of each protein for C, n = 5. **(E)** Immunofluorescence staining for YAP in CHs treated with nocodazole (N) or docetaxel (D) for 1 week. Enclosed areas are enlarged in below panels. Scale bar, 100 μm. **(F)** Quantification of YAP in nucleus. n = 5. MT stabilization inhibited the YAP activity through different regulation in CHs. **(G and H)** RT-qPCR analysis of *MST1, MST2, LATS1, LATS2, RHOA, ROCK1*, and *ROCK2* in CHs treated with either nocodazole (N) or docetaxel (D) for 1 week. Microtubule stabilization promoted the Hippo pathway and inhibited the Rho pathway in CHs. Data are represented as the mean ± SD. *P < 0.05, **P < 0.01, ***P < 0.001.

Yes, associated protein (YAP) is an important mechano-transduction mediator that negatively regulates CHs differentiation 21, 22. We compared the expression of *YAP* and the genes related to YAP signaling pathway in each subpopulation between damaged and intact cartilage (Figure S9B). In the CHI3L1^+^C, the expression of *YAP* was in slightly decreased in the damaged group. The *MST1* was enhanced and the *ROCK1* was repressed in the pathological condition. Following its transfer to the cytoplasm and subsequent phosphorylation, YAP remains in the inactive form. Three types of human OA cartilage specimens were selected to measure the expression of Ace-tubulin and YAP (Figure S9C). In the intact cartilage, Ace-tubulin and YAP expression was negligible. In the second specimen, which showed degeneration but not severe degradation, the Ace-tubulin and cytoplasmic YAP levels were high. Diminished Ace-tubulin and YAP levels were found in the nucleus and degenerated cartilage. In the model rats with cartilage injury, sporadic expression of YAP was found in the damaged group and in the cytoplasm of the repaired cartilage in the docetaxel group (Figure 5A). Moreover, the phosphorylation and expression levels of YAP were measured *in vitro*. Docetaxel treatment significantly increased the phosphorylation rate of YAP without affecting its total protein levels in CHs (Figure 5C-D). Immunofluorescence staining revealed that YAP was predominantly located in the cytoplasm in the CHs of the docetaxel treatment group (Figure 5E and 5F). Furthermore, the mRNA levels of the upstream signaling components of YAP were examined; specifically, the levels of Hippo pathway (*MST1/2* and* LATS1/2*), which repress YAP activity and Rho pathway (*RHOA* and* ROCK1/2*), which promote YAP activity, were measured. The mRNA levels of *LATS1* were increased, whereas the levels of *RHOA, ROCK1* and *ROCK2* were decreased in the docetaxel-treated CHs (Figure 5G-H). These findings indicated that MT stabilization inhibited YAP activity in CHs.

**Figure 6:**
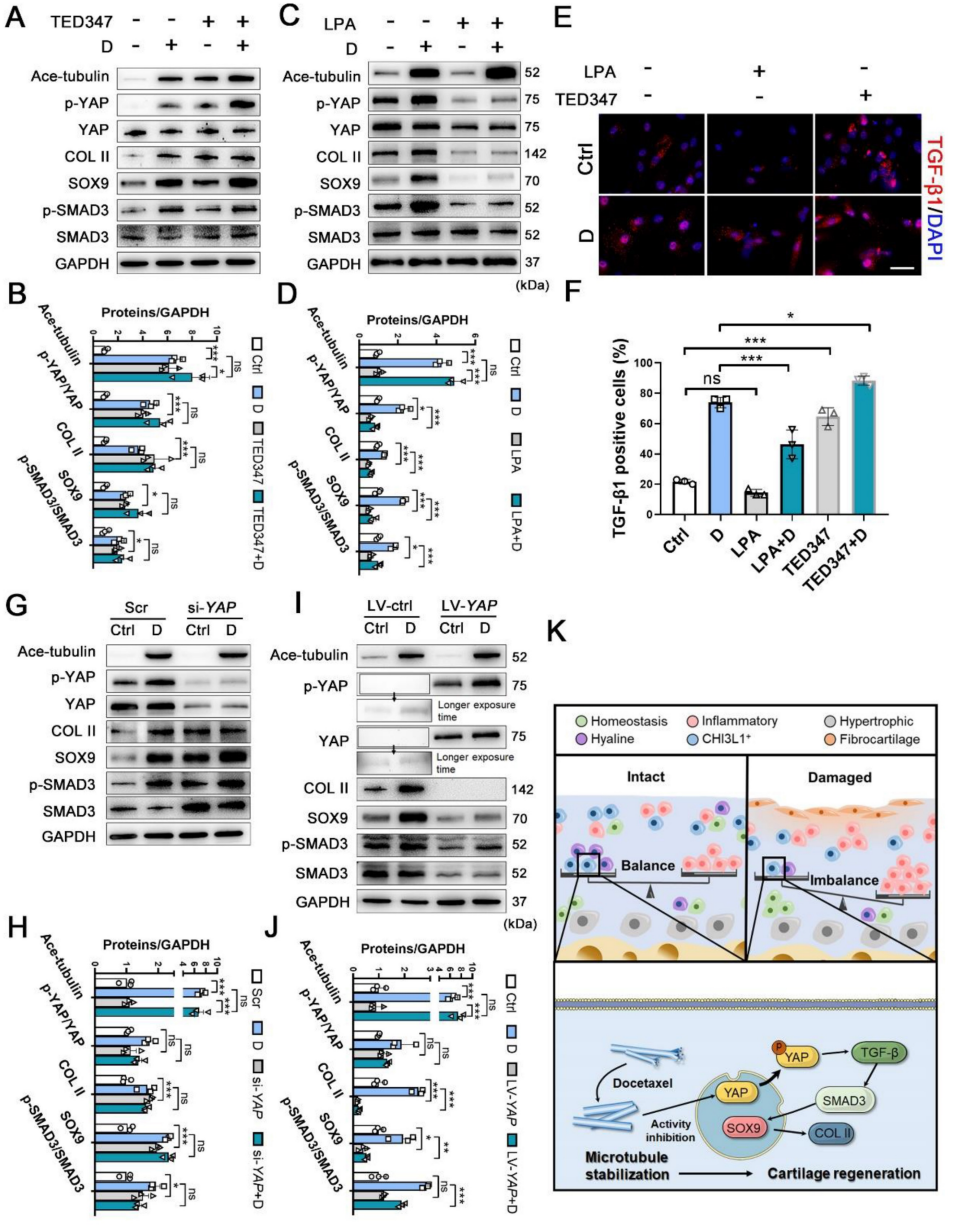
** The effect of inhibition and activation of YAP on chondrogenesis and schematic of high-resolution architecture of human osteoarthritis degeneration and regeneration. (A)** Western blot analysis of Ace-tubulin, phosphorylated YAP, YAP, Col II, SOX9, phosphorylated SMAD3, and SMAD3 in chondrocytes (CHs) treated with TED347 with or not with docetaxel (D) for 1 week. **(B)** Quantification of data A. n = 3. **(C)** Western blot analysis of Ace-tubulin, phosphorylated YAP, YAP, Col II, SOX9, phosphorylated SMAD3 and SMAD3 in CHs treated with LPA with or not with docetaxel (D) for 1 week. **(D)** Quantification of data C. **(E)** Immunofluorescence staining for TGF-β1 in CHs treated with TED347 and LPA independently, with or not with docetaxel (D) (upper panel). **(F)** Quantification of the result of immunofluorescence staining (lower panel), n = 3. **(G)** Western blot analysis of Ace-tubulin, phosphorylated YAP, YAP, Col II, SOX9, phosphorylated SMAD3, and SMAD3 in chondrocytes (CHs) transfected with siRNA-*YAP* with or not with docetaxel (D) for 1 week. **(H)** Quantification of data A, n = 3. **(I)** Western blot analysis of Ace-tubulin, phosphorylated YAP, YAP, COL II, SOX9, phosphorylated SMAD3, and SMAD3 in CHs transfected with overexpress lentivirus-*YAP* with or not with docetaxel (D) for 1 week. **(J)** Quantification of data C, n = 3. **(K)** Schematic of six CHs subpopulation and the balance (intact) and imbalance (damaged) of this CHs involved in degeneration and regeneration in OA cartilage. The mechanism of CHI3L1^+^C involved in the regeneration with MT stabilization by enhancing YAP cytoplasmic translocation in CHs to promote cartilage maintenance. All data are represented as the mean ± SD. *P < 0.05, **P < 0.01, ***P < 0.001.

To determine the role of YAP activity in chondrogenesis and cartilage regeneration, TED347 was utilized to inhibit the combination of YAP with its downstream protein-TEAD. Lysophosphatidic acid (LPA) was used to promote the nucleus re-localization of YAP. The levels of phosphorylated YAP were increased by the TED347 treatment without affecting YAP expression (Figure 6A-B). In addition, the expression levels of SOX9 and COL II were increased. TGF-β1 and SMAD3 were activated following TED347 treatment (Figure 6A-B and 6E-F). However, the difference in the expression levels of these proteins was not significant following TED347 treatment when the CHs were incubated with docetaxel. The level of phosphorylated YAP was significantly decreased following LPA treatment, while SOX9 and COL II expression levels were decreased. The TGF-β1 and SMAD3 were activated, whereas Ace-tubulin levels remained unaffected (Figure 6C-D and 6E-F). Moreover, we investigated the role of YAP expression in chondrogenesis. YAP expression was knocked down using siRNA and overexpressed by lentiviral transfection. The total YAP level was significantly decreased following transfection with *YAP* siRNA with or without docetaxel treatment. In addition, the decrease of YAP, SOX9, COL II and SMAD3 expression was significantly exacerbated (Figure 6G-H). In contrast to these findings, the expression of all these chondrogenesis-related proteins was inhibited when YAP was overexpressed (Figure 6I-J). These findings suggest that YAP is a downstream effector of MT remodeling in CHs and plays a negative role in chondrogenesis and CH development.

## Discussion

Inadequate knowledge of cartilage regeneration poses a challenge to the development of ideal treatment strategies. In this study, we compared the cell subpopulations and networks between damaged and intact cartilage, and a severe imbalance between degeneration and regeneration within CH subpopulations in OA cartilage were identified (Figure 6K). However, we initially found that a CHs subpopulation marked with CHI3L1, exhibited regenerative potency under pathological conditions. According to a gene sets analysis, the CHI3L1^+^C was enriched in cytoskeleton process, notably, in association with MT stabilization. By assessing further relevant biological functions, we verified that MT stabilization indeed potentiated cartilage regeneration and exerted a significant effect on chondrogenesis, via inhibiting YAP activity in CHs.

In recent years, the pathological mechanism about cartilage degeneration in the OA context has been explored. Ji et al. identified seven populations of CHs on the basis of molecule expression in human OA cartilage and revealed their molecular programs and lineage progression patterns 4. Sebastian et al. isolated CHs from adult mouse knee joints before and after traumatic injury and determined the injury-induced molecular changes in the cell subpopulation 23. Lv et al. recently reported a CHs cluster characterized by preferentially expressed ferroptotic genes, namely ferroptotic CHs subpopulation, which played an important role in OA progression 5. In the present study, we focused on the CHs derived from relatively intact and damaged cartilage tissues located in the OA human femoral condyle. By comparing the transcriptome results of the CHs in the two types of cartilage, a specific imbalance in cell clusters was noted in damaged cartilage, which was caused by an increase in the inflammatory cell subpopulation and disruption to the proliferation of the anabolic and regenerative cells, which was consistent with previous studies. The inflammatory phenotypes in the InflamC-1&2 were further amplified and could influence the total cluster in the injured cartilage. It was suggested that the drastic activity of InflamC was the main reason that contributed to the continuous deterioration of cartilage function. RPS4Y1^+^CHs (HyC) were responsible for cartilage anabolism and functional maintenance. Severe abrasions to cartilage resulted in a reduction in the cell number and the disruption of the anabolic capability of the HyC. The characterization of these CH populations in pathological situations improved our understanding of cartilage degeneration in human OA. However, until now, the cartilage regeneration in different CHs subpopulations was not clearly understood.

Cartilage disease has been considered a troublesome disorder because of poor intrinsic regeneration 3, which is the main obstacle to successful clinical therapy for cartilage injury. Although the catabolism level was markedly increased in the degenerative cartilage, the expression levels of SOX9 and COL II were upregulated, which indicated spontaneous regeneration and constrained cartilage self-healing. The present study aimed to increase the understanding of regenerative cell subpopulations in the pathological cartilage. Previous studies have shown that chondrogenic progenitor cells (CPCs) exhibit multi-lineage differentiation potential and reparative capacity in cartilage tissues 24, 25. In a study comparing osteoarthritic and healthy cartilage by single cell sequencing, CPCs were mainly located in the middle and deep zones of normal healthy cartilage with highly expressed genes related to self-renewal ability 26. However, CPCs were found to participate in self-repair during the early-stage OA 24, 25, 27. The mechanisms of cartilage regeneration in the progression of OA or damaged cartilage remain largely unknown. Surprisingly, the present study identified a cell subpopulation, labelled CHI3L1, with cartilage regenerative potential and stem cell phenotypes in damaged cartilage. The gene set of CHI3L1^+^C was enriched in regeneration and CH differentiation. Although damage factors and inflammatory signals lessened its cell proportion, CHIL31^+^C exhibited increased cartilage anabolism and an ability to respond to detrimental factors. These results implied that the CHIL31^+^C played a protective role and restored original cartilage function under pathological conditions. Herein, considering the genetic features and molecular network of CHI3L1^+^C, we suggested that this CHs subpopulation can be further named the reactive regeneration CHs (ReRegC). A prior single-cell sequencing analysis of cartilage by Ji et al. 4 led to the identification of a specific population of regulatory CHs. Similar to those in the ReRegCs, the regulatory CHs were exhibited high expression of *CHI3L1.* It was reported that regulatory CHs were significantly enriched with signaling pathway regulating and cellular response genes. In a study of CHs in human intervertebral discs, a CHs cluster with high expression of *CHI3L1* reflected protective characteristics 28. Notably, although chitinase-3-like protein 1 (CHI3L1) was known as a proinflammatory glycoprotein biomarker during cartilage degeneration 29, 30, the overexpression of this inflammatory-sensitive protein decreased the catabolism and increase the anabolism of ECM, playing a protective role in the degeneration of intervertebral disc 31. Taken together, we deciphered the key role of *CHI3L1*-marked ReRegCs with regard to self-compensation and rescue procedures in cartilage regeneration.

We believe that the mechanism involved in ReRegC (CHI3L1^+^C) is an effective treatment target for cartilage injury. We observed that cytoskeleton-related genes and signals were highly expressed in the CHI3L1^+^C. Further GSEA revealed the relationship between this CHs population and the MT process. MTs play key roles in various cellular processes, including maintenance of cell shape, motility, transport and interactions with the ECM. MTs are constantly remodeled through alternating growth and shrinkage of their extremities 12, 32. The status of MTs, which includes unstable and stable forms, determines cell organization, and MTs are involved in motor-driven intracellular transport. Stabilization of MTs protects them from break-down, and further provides a mechanism for responding to signals as well as forming a basic structure for protein transportation and secretion 13. It has been hypothesized that MT stabilization is associated with the regulation of intrinsic regeneration and the cellular response of CHs in disease contexts. Our recent works demonstrated that MT stabilization played an important role in promoting the chondrogenesis of synovial mesenchymal stem cells and inhibiting the fibrosis of fibrotic chondrocytes 33, 34. Herein, MT stabilization increased cartilage anabolic factors and upregulated TGF-β signaling in CHs treated with docetaxel. Subsequently, we used the rat cartilage injury model to directly verify the cartilage regenerating effect of MT stabilization. The increasing proportion of CHI3L1^+^C subpopulation indicated that MT stabilization improved the proportion of the ReRegCs to enhance cartilage regeneration. In generally, MT stabilization-associated drugs are used in the clinic and basic research 13, 14, 19; therefore, the mechanism of MT stabilization might be a potential and practical strategy for therapy in cartilage injury. However, most MT stabilization associated drugs are used for the therapy of tumor and are toxic to many cells when administered at high concentration 19. Our results showed that the cell viability was reduced when docetaxel treatment was administered at high doses. Thus, attention needs to be focused on the exploring the optimal approach for drug release at safe and efficient concentrations. Moreover, there are many microtubule-binding proteins (MTBPs) on microtubules that regulate microtubule aggregation and depolymerization 35. MTBP can be classified into microtubule-stabilizing proteins (stabilizers) and microtubule destabilizing protein (destabilizers) based on their functions. Aberrant mechanical force is the main pathological reason of OA. However, the regulatory influence of mechanical force on MTBPs and MTs stabilization in the OA context is still unclear nowadays. More understanding of the role of MTBPs in the cartilage degermation and regeneration is needed to identify therapeutic targets for cartilage disease.

The mechanism of the effect of MT stabilization was involved in the inhibition of YAP activity. YAP has been reported to be an important factor for sensing and transferring mechanical signals 36. The role of YAP signaling in the homeostasis of cartilage varies. A recent study demonstrated that YAP played an important role in the antagonistic effect on NF-κB signaling to resist matrix-degrading enzyme activity and cartilage degradation in OA 37. The activation of YAP signaling also prevented the pre-inflammatory process and repressed the cartilage breakdown via the reduction of expression of primary cilia 38. However, YAP is known to play a negative role in chondrogenesis 22. During the differentiation of neural crest cells *in vitro*, YAP significantly increased the expression of osteogenic genes such as *Runx2* and *Sp7* but inhibited the expression of chondrogenic genes such as *Sox9* and *Col2a1* 39. Meanwhile, connective tissue growth factor (CTGF) has been identified as the key downstream target of YAP 37. Aberrant CTGF function is involved in excessive tissue fibrosis 38, 39. Several studies have reported that YAP played a critical role in fibrogenesis and promoting fibrotic disease progression, including skin scarring, kidney fibrosis and liver fibrosis 40-42. In our previous work, we found that the expression of YAP was directly dampened in synovial mesenchymal stem cells by MT stabilization because of the influence on depolymerization of actin 33. While, we verified that the increasing chondrogenesis caused by MT stability was mainly mediated by inhibition of YAP activity, not the expression of YAP. Therefore, the precise role of YAP and its regulatory effect on the degeneration and regeneration processes of cartilage under pathological conditions need to be further studied. The physiopathology of cartilage remains to be elucidated since specific mechanisms are potentially involved in the interplay between MT stabilization and cytoskeleton-related signaling proteins.

In summary, we described a high-resolution architecture of regeneration processes in human osteoarthritic cartilage and determined its relationship with MT process. The identification of the CHI3L1^+^C subpopulation and its association with MT stabilization highlighted a potential mechanism underlying cartilage regeneration and chondrogenesis. The role of MT stabilization in cartilage repair presents a promising therapeutic target for OA or cartilage injury.

## Materials and Methods

### Ethics statement

The collection of and experimental protocols using human articular cartilage and synovium were approved by the Ethical Committee of the Nanjing Drum Tower Hospital, the Affiliated Hospital of Nanjing University Medical School (2020-156-01). All experimental procedures followed the guidelines established by the Declaration of Helsinki. All surgical operations, treatments and postoperative care procedures for animal studies were performed in strict accordance with the Animal Care and Use Committee of the Nanjing Drum Tower Hospital, the Affiliated Hospital of Nanjing University (2020AE02013).

### Human chondrocyte sample collection

Cartilage specimens were acquired from 3 patients (Kellgren-Lawrence grade IV) undergoing knee arthroplasty surgery at the Nanjing Drum Tower Hospital. The information of the patients was showed in the Table S1 and Figure S1. Samples from the same patients were divided into damaged and intact group. The damaged cartilage samples were captured in the medial femoral condyle with cartilage abrasions. The intact cartilage samples were captured in the in the censure of lateral femoral condyle with intact cartilage surface.

### Single cell suspension preparation

The cartilage tissue was minced into small pieces and digested in 0.2% collagenase II (Sigma-Aldrich, Darmstadt, Germany) at 37 °C for 1 hour. The enzymatic reaction was quenched by 10% fetal bovine serum (Thermo Fisher, New York, United States) in RPMI-1640 and the suspension was filtered through a 70μm strainer to remove the incompletely digested clumps debris. Then the filtered cell suspension was centrifuged, after supernatant removed cell pellets were resuspended in DPBS buffer. After centrifuged and resuspended, the cells were counted and assessed for viability (> 85%) using Countess® II Automated Cell Counter (Thermo Fisher) and 150,00 CHs were re-suspended in Drop-seq loading buffer.

### Single cell library construction and RNA Sequencing

Single-cell suspension was loaded on a Chromium Single Cell Platform using the Chromium Single Cell 3'Library and Gel Bead Kit v2 (10X Genomics, PN-120237) and the Chromium Single Cell A Chip Kit (10X Genomics, PN-120236) as per the manufacturer's protocol. In brief, cell suspensions were added to each lane of the 10X chip. The cells were partitioned into Gel Beads in Emulsion in the Chromium instrument, in which cell lysis and bar-coded reverse transcription of RNA occurred, followed by amplification, fragmentation and 5'adaptor and sample index attachment. Libraries were sequenced on an Illumina HiSeq Xten.

### Pre-processing of single-cell RNA-Seq data

We aligned to the GRCh38 reference genomes as appropriate for the input dataset, and estimated cell-containing partitions and associated unique molecular identifiers (UMIs), using the Cell Ranger v.3.0.1. For quality control, we excluded cells in which less than 560 genes or more than 8085 genes were detected. Cells with less than 1,000 total counts or more than 76010 total counts or mitochondrial gene content >10% of the total UMI count were also filtered. Genes that are detected in more than 10 cells were kept. After quality control, 20839 cells were kept.

### Dimension reduction, clustering and differential gene expression analysis

After obtaining the qualified expression data matrix, we used Seurat 43 (v.3.2.3) for dimension reduction, clustering and differential gene expression analysis. We used shared nearest neighbour graph-based clustering, in which the graph was constructed using from 1 to 25 principal components as determined by dataset variability shown in principal component analysis (PCA); the resolution parameter to determine the resulting number of clusters was also tuned accordingly. Genes significantly enriched in each cell cluster were identified using the FindAllMarkers function with default parameters in Seurat. Subsequent functional annotation of the marker genes was performed using Metascape 44.

### Single-cell trajectory construction

Single-cell trajectory was performed on cell clusters in cartilage using the Monocle package (v2.16.0) 45. 629 Differentially expressed genes between each cluster was selected as the ordering genes. We choose 2 for the max_components and 'DDRTree' for the reduction method when performing the reduceDimension function.

### Analyzing cellular interactions

To systematic analysis the inter-cellular interactions, we used CellPhoneDB 15 and Cellchat 46. We considered only ligands and receptors expressed in greater than 10% of the cells in any given subpopulation.

### Gene set analysis

Gene set enrichment analysis (GSEA) analysis was performed by clusterProfiler package (version 3.16.0). The differential expressed genes and the average LogFC between the injured and uninjured cells of cluster4 were used as inputs. Gene set variation analysis (GSVA) was performed by the GSVA R-package (version 1.38.2). Gene-by-cell matrix is converted into a gene-set-by-cell matrix firstly. GSVA scores were calculated for sets with a minimum of 5 detected genes. All other parameters were default. Only significant GO terms were displayed in the heatmap.

### Cell culture

Human OA cartilage samples (n=7) were processed to establish primary cell cultures. Fresh cartilage was cut into 1 mm^3^ cubes or fragments respectively and were washed with phosphate-buffered saline (PBS). The cartilage cubes were lysed with 0.2% collagenase II in Dulbecco's modified Eagle medium/nutrient mixture F12 (DMEM/F12) at 37 °C for 6 h. The cells were cultivated in a humidified environment at 37 °C and 5% CO_2_ followed by regular replacement of the culture medium every two days.

### Magnetic-activated cell sorting (MACS)

After preparing the primary cells, the cells were stained with primary CD79B antibody (anti-human, PE, REAfinity) (1:50, Miltenyi Biotech, Bergisch Gladbach, Germany) and incubated with anti-PE MicroBeads (1:10, Miltenyi Biotech) subsequently. The cell suspension was applied into the MACS column, and the CD79B^-^ cells that passing through column was collected with appropriate amount of column wash buffer. After removing column from the separator, the magnetically labeled cells (CD79B^+^) was collected from column.

### Human samples for biological analysis

Human OA cartilage were collected from 15 OA patients (Kellgren-Lawrence grade IV) when they underwent total knee arthroplasties. Four samples were processed for cartilage protein analysis, one was processed for histological examination, and the remaining were processed to establish primary cultures of CH.

### Microarray Data Collection and Processing

A gene expression profile (GSE16464) was downloaded from the Gene Expression Omnibus (GEO) database (http://www.ncbi.nlm.nih.gov/geo/). The microarray dataset 18 was based on HGU133plus2.0 platform and contained articular cartilages from three OA donors and three healthy donors. Raw data were normalized by robust multi-array average (RMA) algorithm. There were 54,675 probes and combined into 20,161 gene symbols. Tubulin related genes were extracted and filtered by |log(Fold Change)| > 1.25 with *P* value < 0.05. Heatmap and GO dotplot were conducted with R pheatmap (v1.0.12), gglpot2 (v3.32) packages.

### Quantitative real-time PCR

Cellular mRNA was isolated from CHs using TRIzol reagent (Thermo Fisher, Logan, UT, USA). Complementary DNA (cDNA) was synthesized from mRNA using reverse transcription reagents (Vazyme Biotech, Nanjing, China) and quantitative PCR assays were carried out using a LightCycler®480 II (Roche Molecular Biochemicals, Indianapolis, IN, USA). The primer sequences are listed in Table S2.

### Western blot analysis

Protein was extracted from the cells using RIPA lysis buffer supplemented with 1 mM phenylmethanesulfonyl fluoride and 1 mM protein phosphatase inhibitor. The protein concentration of the samples was determined by BCA protein assay kit (Thermo Scientific). Proteins from the prepared lysates were then separated on 10% (w/v) SDS-polyacrylamide gels and transferred onto a polyvinylidene fluoride membrane (Bio-Rad, Hercules, CA, USA). The membranes were blocked with 5% (w/v) milk (Bio-Rad) for 2 h at room temperature and then incubated overnight at 4°C with primary antibodies. The information of primary antibodies used were listed in Table S3. The membranes were then washed using TBS with 0.05% Tween 20 (TBST) and incubated with horseradish peroxidase-conjugated secondary antibodies for 60 min. All protein signals were detected using a ChemiDocXRS + Imaging System (Tanon, Shanghai, China). All experiments were repeated 5 times.

### Animal study

To study the effect of MT stabilization on cartilage injury, adult male Sprague Dawley (SD) rats (220-250 g, n=20) were acquired from the Animal Center of the Nanjing Medical University (Jiangsu, China) and acclimated for 1 week prior to the operation. After acclimation, full-thickness cartilage defects (2.0 mm in diameter, 3.0 mm in depth) were established in the center of the trochlear groove in right knee of 15 rats using an osteochondral transplantation instrument and five rats received a sham operation. After surgery, the rats were divided into four groups (n=5 each): the sham, control (cartilage injury), nocodazole (cartilage injury + nocodazole treatment), and docetaxel groups (cartilage injury + docetaxel treatment). Nocodazole (2 μg/kg in physiological saline) (Sigma-Aldrich), docetaxel (50 μg/kg in physiological saline) (Aladdin, Shanghai, China), or physiological saline was delivered to rats with cartilage injury via intra-articular injection on a weekly basis starting at one week after surgery and continuing until the rats were sacrificed at six weeks after surgery.

### Histological and microscopy analysis

Cartilage tissues from animals and human OA patients were fixed in 4% (v/v) paraformaldehyde for one day and then decalcified in 10% (v/v) EDTA for two months. After dehydration, the specimens were embedded in paraffin and cut into 3-μm coronal sections. Sections of each tissue were then processed and stained with safranin O-fast green (SO) and hematoxylin and eosin (H&E). The appearance of cartilage defects in the femoral trochlea from rats in each experimental group were evaluated according to the International Cartilage Repair Society (ICRS) macroscopic evaluation scoring system 47. The histological staining results for each experimental group were evaluated according to the ICRS visual histological evaluation scoring system 48.

### Immunohistochemical staining and immunofluorescent analysis

Immunohistochemical staining and immunofluorescent analysis were performed according to the manufacturer`s instructions. Serial sections were incubated with primary antibodies overnight at 4 °C. The information of primary antibodies used were listed in Table S3. For immunohistochemical staining, HRP conjugated secondary antibodies were added to the slides and incubated at 37 °C for 1 h. For immunofluorescent staining, FITC or TRITC conjugated secondary antibodies were added to the slides and incubated at room temperature for 1 h in the dark. Photomicrographs of sections were captured with a fluorescence microscope (Zeiss, Heidelberg, Germany).

### Pellet cultures

Pellets consisting of 5 × 10^5^ cells were cultured in microfuge tube for 4 weeks in a humidified environment at 37 °C and 5% CO_2_ with chondrogenic differentiation medium. The medium was supplemented with 10 ng/mL TGF-β1 (Peprotech, New Jersey, USA) and 500 ng/ml BMP2 (Peprotech). The medium was refreshed twice per week.

### Short interfering RNA (siRNA) transfection

siRNA against the human *YAP1* gene was designed and synthesized as the following sequence: 5-GGUGAUACUAUCAACCAAATT-3 (Hippobio). CHs were seeded in 6-well plates and grown to approximately 70% confluence. Cells were then transfected with either 50 nM siRNA-YAP or negative control in Lipofectamine 3000 for 12 h (Thermo Fisher) according to the manufacturer's instructions.

### Statistical analysis

All data were expressed as means ± SD. Statistical analysis was performed with one-way analysis of variance (ANOVA) using GraphPad Prism 8 for Windows. Quantitative data represents at least three independent experiments. Shapiro-Wilk test and Levene method were used for the estimation of the data normal distributions and homogeneity of variance, respectively. For the comparison of mean values between two groups, paired or unpaired two-tailed Student's t test was used. One-way analysis of variance (ANOVA) followed by Tukey's post hoc tests were used to assess the statistical significance of the mean values of more than two groups. Differences were considered statistically significant when P < 0.05.

## Supplementary Material

Supplementary figures and table.

## Abbreviations

CHs: Chondrocytes
OA: Osteoarthritis
MT: Microtubule
ECM: Extracellular matrix
COL II: Type II collagen
COL I: Type I collagen
TKA: Total knee arthroplasty
t-SNE: t-distributed stochastic neighbor embedding
HomC: Homeostasis chondrocytes
HyC: Hyaline chondrocytes
HyperC: Hypertrophic chondrocytes
InflamC: Inflammatory chondrocytes
FibroC: fibrocartilage chondrocytes
GSEA: Gene set enrichment analysis
GO: Gene Ontology
KEGG: Kyoto Encyclopedia of Genes and Genomes
TGF-β: Transforming growth factor
MACS: magnetic-activated cell sorting
CM: chondrogenic medium
YAP: yes associated protein
CPCs: Chondrogenic progenitor cells
ReRegC: Reactive regeneration chondrocytes
CTGF: connective tissue growth factor
