# Supplementary material for: Microtubule stabilization targeting regenerative chondrocyte cluster for cartilage regeneration
Source: Theranostics. 2023 Jun 12;13(10):3480–96. doi: 10.7150/thno.85077 (PMC10283062; doi:10.7150/thno.85077)
Supplement: Supplementary file 1 — Supplementary figures and table. [file thnov13p3480s1.pdf]

Supplementary information

Microtubule stabilization targeting regenerative chondrocyte cluster for cartilage regeneration

Jiawei Li[1,3†], Chunmei Fan[2,4,5,8,9†], Zhongyang Lv[1†], Ziying Sun[1], Jie Han[2,5,8,9], Maochun Wang[1], Huiming Jiang[6], Kuoyang Sun[1], Guihua Tan[1], Hu Guo[1], Anlong Liu[1], Heng Sun[1], Xingquan Xu[1], Rui Wu[1], Wenjin Yan[1], Qing Jiang[1], Shiro Ikegawa[1,7], Xiao Chen[2,4,8,9*], Dongquan Shi[1,9*]

	Patient 1	Patient 2	Patient 3
Diagnose	Right knee OA	Right knee OA	Left knee OA
Gender	Male	Female	Female
Age (years old)	82	64	75
Weight (kg)	74	58	54

Table S1. The information of human OA cartilage samples for Single-cell RNA sequence.

SOX9	Forward primer	5’-AGCGAACGCACATCAAGAC-3’
	Reverse primer	5’-CTGTAGGCGATCTGTTGGGG-3’
COL2A1	Forward primer	5’-CCAGATGACCTTCCTACGCC-3’
	Reverse primer	5’- TTCAGGGCAGTGTACGTGAAC-3’
RUNX2	Forward primer	5’- TGGTTACTGTCATGGCGGGTA-3’
	Reverse primer	5’- TCTCAGATCGTTGAACCTTGCTA-3’
COL1A1	Forward primer	5’- GAGGGCCAAGACGAAGACATC-3’
	Reverse primer	5’- CAGATCACGTCATCGCACAAC-3’
COL10A1	Forward primer	5’- ATGCTGCCACAAATACCCTTT-3’
	Reverse primer	5’- GGTAGTGGGCCTTTTATGCCT-3’
MST1	Forward primer	5’- CAGAGCTGCGGCATCAAATC-3’
	Reverse primer	5’- ACCTTGGTCGAGGAACTTGC-3’
MST2	Forward primer	5’- TCTGAGATTGTGGAGGCCATTC-3’
	Reverse primer	5’- GCTCCGTTCCTAAGGCAGAT-3’
LATS1	Forward primer	5’- CTGCTCTCCCCTCCAGAGTTA-3’
	Reverse primer	5’- TGGCAGGAAAGGTCTTAGGC-3’
LATS2	Forward primer	5’- TGCCAACAATGTAGCGAATGT-3’
	Reverse primer	5’- TTGAAGATTATCACTCTCTCCAGG-3’
RHOA	Forward primer	5’- GATTGGCGCTTTTGGGTACAT-3’
	Reverse primer	5’- AGCAGCTCTCGTAGCCATTTC-3’
ROCK1	Forward primer	5’- AAGAGGGCATTGTCACAGCA-3’
	Reverse primer	5’- AGCATCCAATCCATCCAGCA-3’
ROCK2	Forward primer	5’- CCCGATAACCACCCCTCTTC-3’
	Reverse primer	5’- TGCCTTGTGACGAACCAACTG-3’
GAPDH	Forward primer	5’-ACAACTTTGGTATCGTGGAAGG-3’
	Reverse primer	5’- GCCATCACGCCACAGTTTC-3’

Table.S2 The primer sequences used in RT-PCR analysis

Primary Antibody	Concentration	Supplier
For Western blot		
Ace-tubulin	1:1000	Cell Signaling Technology, Boston, USA
Col II	1:5000	Abcam, Cambridge, UK
CD79B	1:500	Proteintech, Wuhan, China
SMAD3	1:1000	Cell Signaling Technology
Phosphorylated SMAD3	1:1000	Cell Signaling Technology
YAP	1:1000	Cell Signaling Technology
Phosphorylated YAP	1:1000	Cell Signaling Technology
GAPDH	1:1000	Cell Signaling Technology
For Immunofluorescent and immunohistochemical staining		
Col II	1:500.	Abcam
Col I	1:300	Boster, Wuhan, China
Ace-tubulin	1:500	Cell Signaling Technology
TGF-β1	1:200	Proteintech
CHI3L1	1:200	Proteinech
YAP	1:500	Cell Signaling Technology

Table.S3 The information of primary antibody used in western blot and histological analysis.

Fig. S1

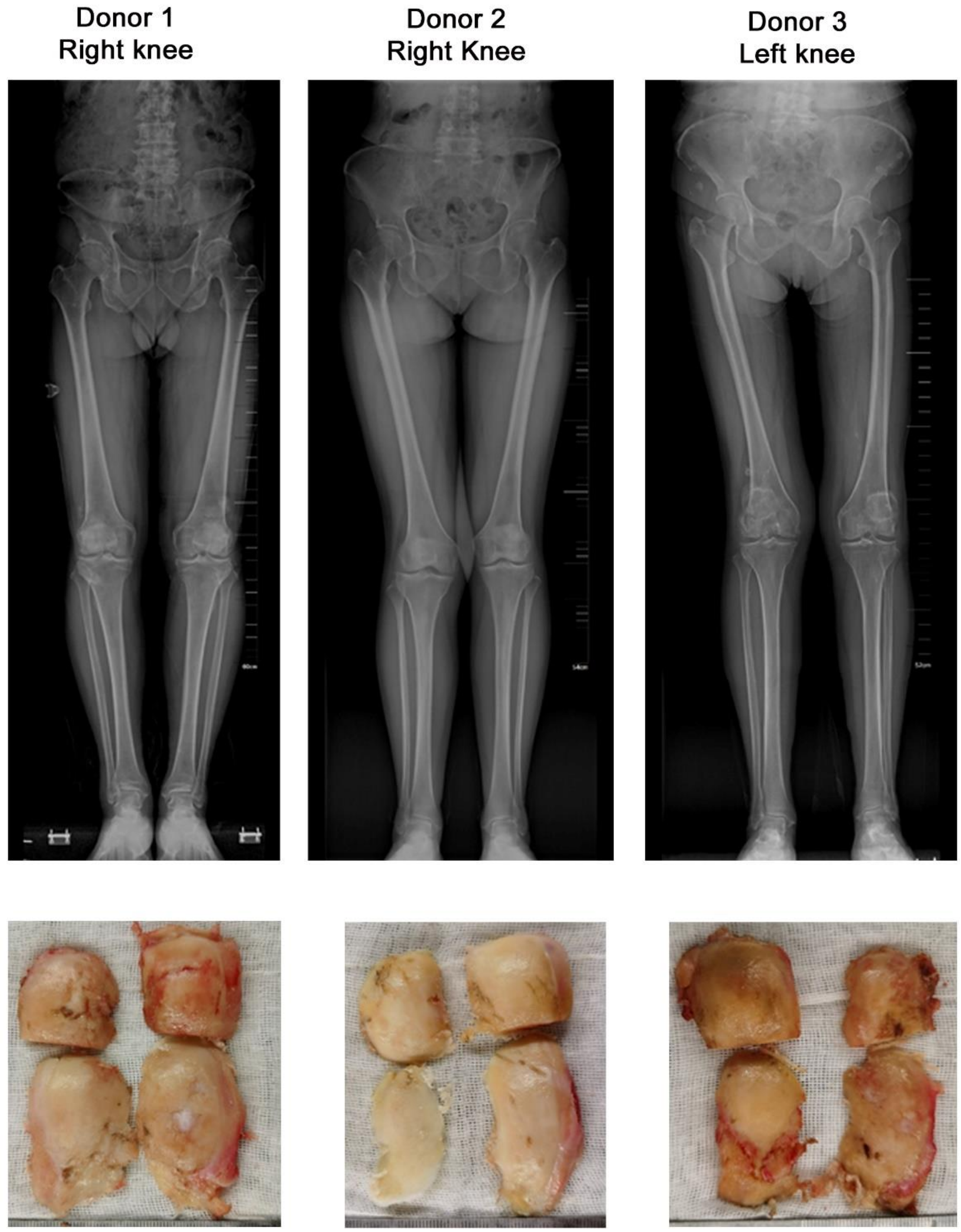

Fig.S1. The radiographic images and general view of the osteoarthritic cartilage form 3 donors

Fig.S2

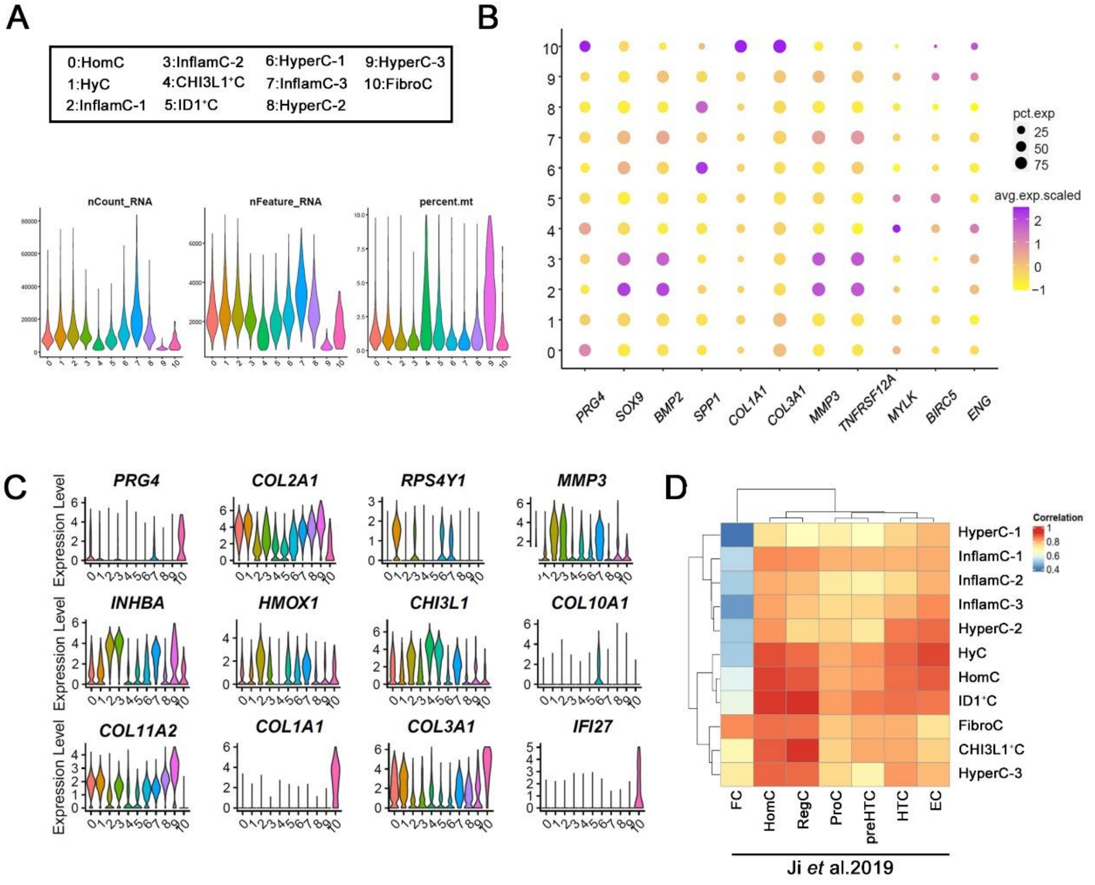

Fig S2. Single-cell atlas of human articular chondrocyte.

(A) Violin plots showing the distribution of the total counts, number of the genes, and the percentage of mitochondrial genes per cell cluster. The CHs clusters represented by number were listed at the right panel. **(B)** Dot plot showing the expression of the representative lineage-related genes of each cluster. **(C)** Violin plots showing expression levels of the represented marker genes of chondrocyte (CH) clusters. **(D)** Heatmap showing pairwise Pearson correlations in the global transcriptome between CHs in this study and articular chondrocytes based on the research of Ji *et* al(4). FC: fibrocartilage chondrocyte, HomC: homeostatic chondrocyte, HTC: hypertrophic chondrocyte, preHTC: prehypertrophic chondrocyte, ProC: proliferative chondrocyte, RegC: regulatory chondrocyte, EC: effector chondrocyte.

Fig.S3

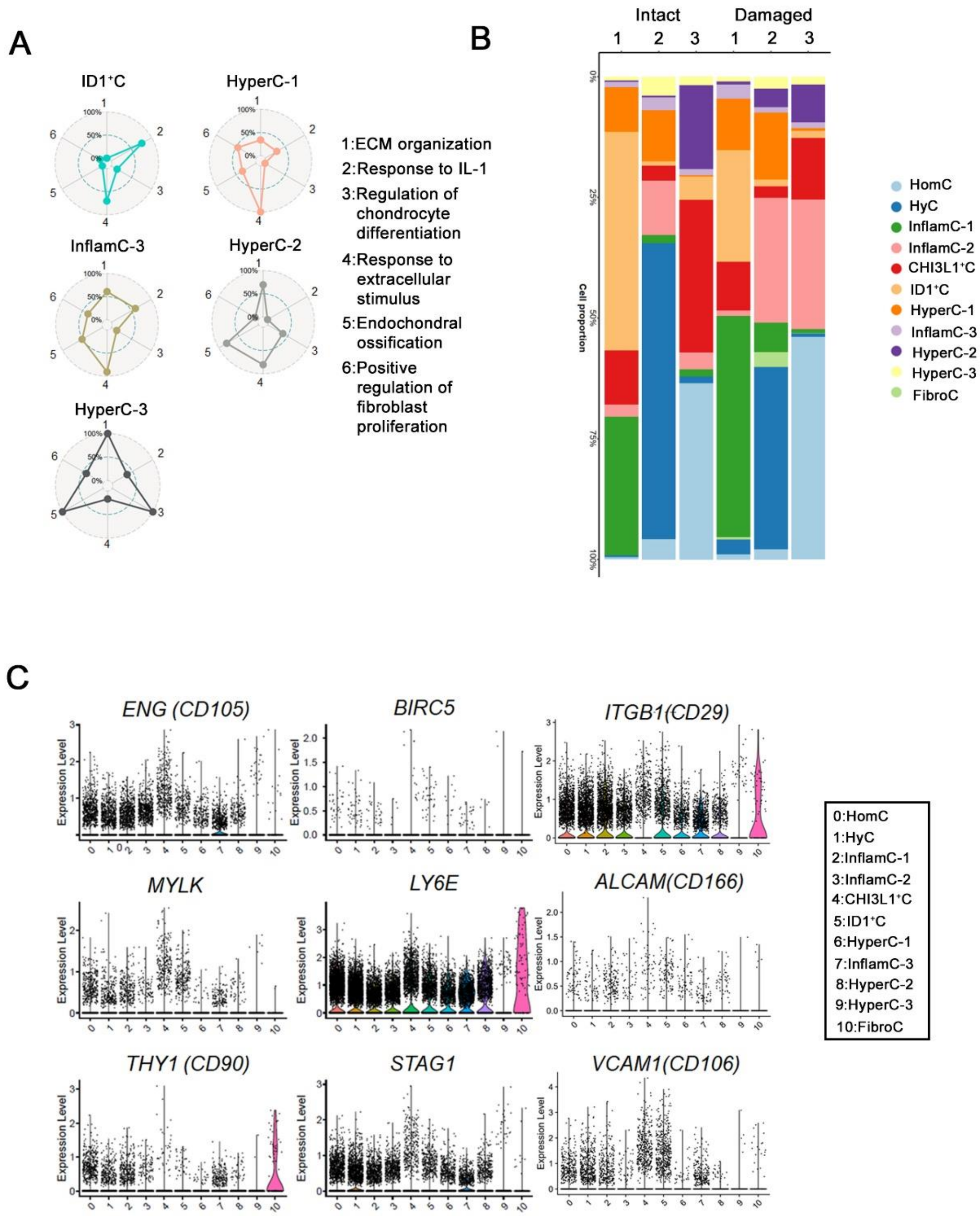
A
ID1⁺C
HyperC-1
InflamC-3
HyperC-2
HyperC-3
1:ECM organization
2:Response to IL-1
3:Regulation of chondrocyte differentiation
4:Response to extracellular stimulus
5:Endochondral ossification
6:Positive regulation of fibroblast proliferation
B
Intact
Damaged
Cell proportion
HomC
HyC
InflamC-1
InflamC-2
CHI3L1⁺C
ID1⁺C
HyperC-1
InflamC-3
HyperC-2
HyperC-3
FibroC
C
ENG (CD105)
BIRC5
ITGB1(CD29)
MYLK
LY6E
ALCAM(CD166)
THY1 (CD90)
STAG1
VCAM1(CD106)
Expression Level
0:HomC
1:HyC
2:InflamC-1
3:InflamC-2
4:CHI3L1⁺C
5:ID1⁺C
6:HyperC-1
7:InflamC-3
8:HyperC-2
9:HyperC-3
10:FibroC

Fig S3. Proportion of the subpopulation in human articular chondrocyte and the stem cell genes expression in each cluster.

(A) Radar map showing the performance of six gene sets associated with the indicated function among each chondrocyte (ID1$^+$C, HyperC-1, Inflam-3, HyperC-2, and HyperC-3). **(B)** Cell proportion of each cell clusters in each human cartilage samples. **(C)** Violin plots showing expression levels of the represented stem cell related genes of each CH cluster.

Fig.S4

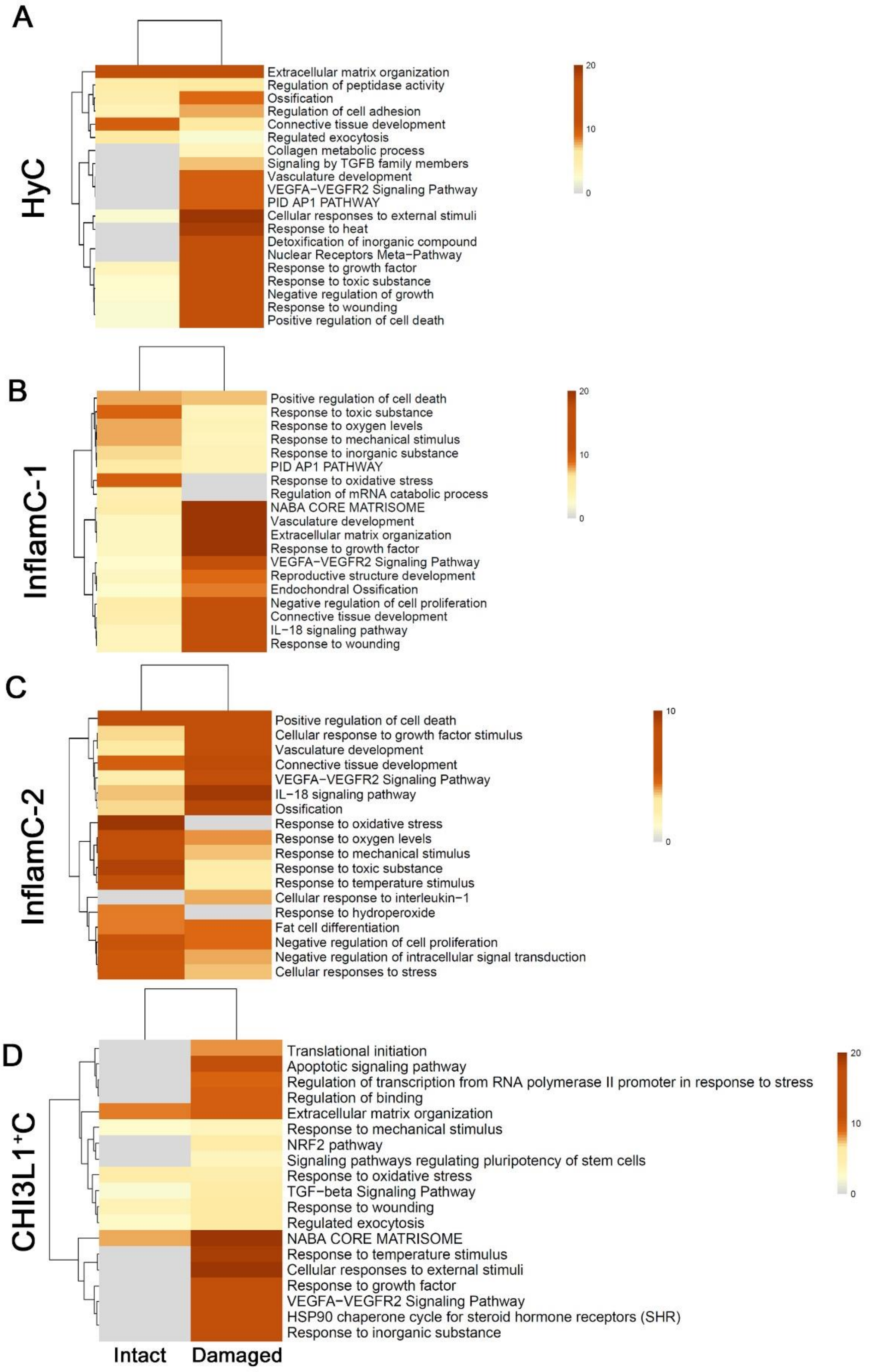
A
HyC
Extracellular matrix organization
Regulation of peptidase activity
Ossification
Regulation of cell adhesion
Connective tissue development
Regulated exocytosis
Collagen metabolic process
Signaling by TGFB family members
Vasculature development
VEGFA−VEGFR2 Signaling Pathway
PID AP1 PATHWAY
Cellular responses to external stimuli
Response to heat
Detoxification of inorganic compound
Nuclear Receptors Meta−Pathway
Response to growth factor
Response to toxic substance
Negative regulation of growth
Response to wounding
Positive regulation of cell death
20
10
0
B
InflamC-1
Positive regulation of cell death
Response to toxic substance
Response to oxygen levels
Response to mechanical stimulus
Response to inorganic substance
PID AP1 PATHWAY
Response to oxidative stress
Regulation of mRNA catabolic process
NABA CORE MATRISOME
Vasculature development
Extracellular matrix organization
Response to growth factor
VEGFA−VEGFR2 Signaling Pathway
Reproductive structure development
Endochondral Ossification
Negative regulation of cell proliferation
Connective tissue development
IL−18 signaling pathway
Response to wounding
20
10
0
C
InflamC-2
Positive regulation of cell death
Cellular response to growth factor stimulus
Vasculature development
Connective tissue development
VEGFA−VEGFR2 Signaling Pathway
IL−18 signaling pathway
Ossification
Response to oxidative stress
Response to oxygen levels
Response to mechanical stimulus
Response to toxic substance
Response to temperature stimulus
Cellular response to interleukin−1
Response to hydroperoxide
Fat cell differentiation
Negative regulation of cell proliferation
Negative regulation of intracellular signal transduction
Cellular responses to stress
10
0
D
CHI3L1+C
Translational initiation
Apoptotic signaling pathway
Regulation of transcription from RNA polymerase II promoter in response to stress
Regulation of binding
Extracellular matrix organization
Response to mechanical stimulus
NRF2 pathway
Signaling pathways regulating pluripotency of stem cells
Response to oxidative stress
TGF−beta Signaling Pathway
Response to wounding
Regulated exocytosis
NABA CORE MATRISOME
Response to temperature stimulus
Cellular responses to external stimuli
Response to growth factor
VEGFA−VEGFR2 Signaling Pathway
HSP90 chaperone cycle for steroid hormone receptors (SHR)
Response to inorganic substance
20
10
0
Intact
Damaged

Fig S4. Enriched Gene Ontology terms of chondrocytes

(A-D) Heatmap showing the enriched Gene Ontology((biological processes)) terms of DEGs between intact and damaged chondrocytes of HyC(A), InflamC-1(B), InflamC-2(C), and ReRegC (D).

Fig.S5

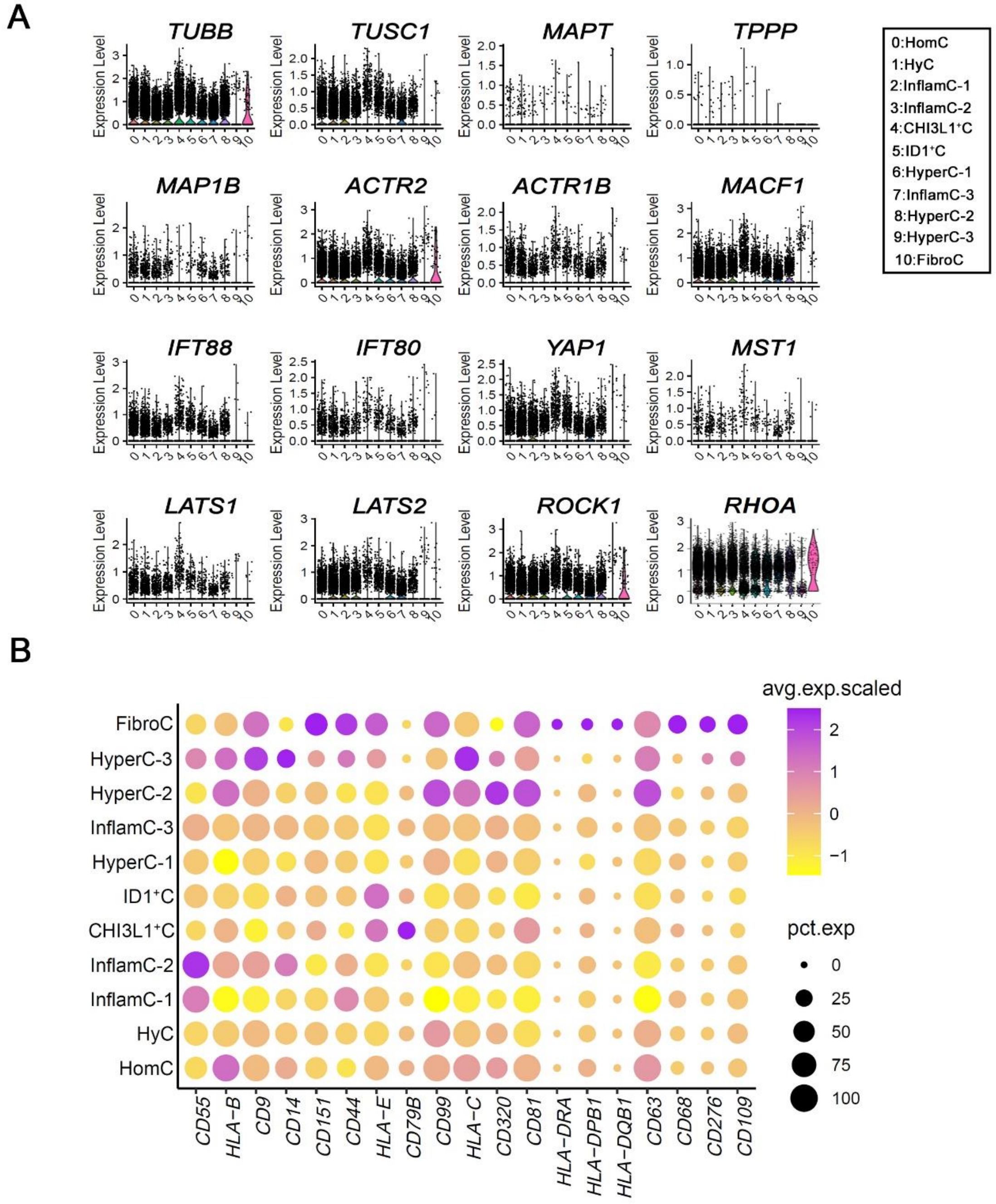

Fig S5. The relationship between cytoskeleton with chondrocytes and osteoarthritis

(A) Violin plots showing expression levels of the represented MT and actin genes of each CH cluster. **(B)** Dot plots showing the expression of cell surface markers in each clusers.

Fig.S6

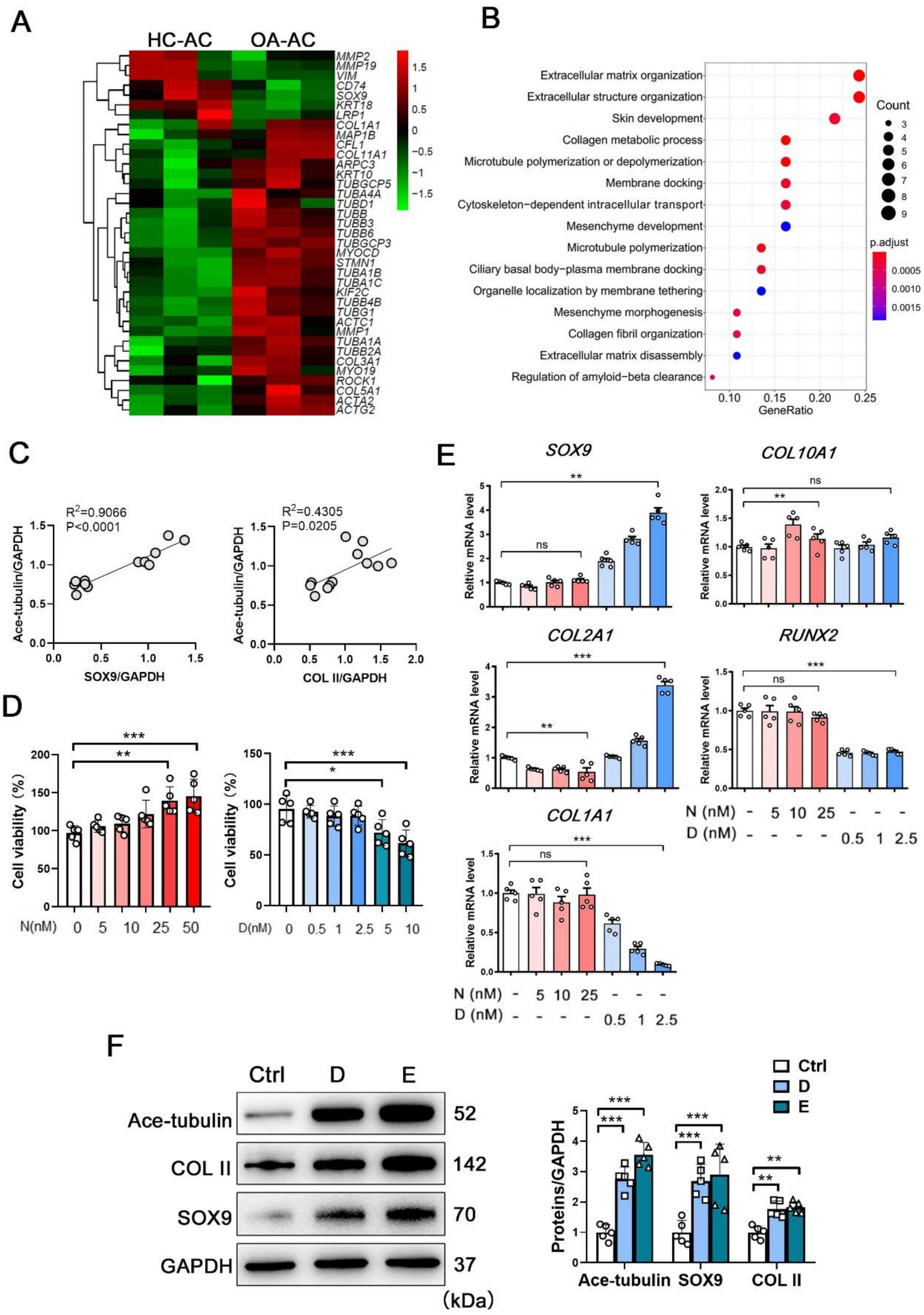
A
HC-AC
OA-AC
MMP2
MMP19
VIM
CD74
SOX9
KRT18
LRP1
COL1A1
MAP1B
CFL1
COL11A1
ARPC3
KRT10
TUBGCP5
TUBA4A
TUBD1
TUBB
TUBB3
TUBB6
TUBGCP3
MYOCD
STMN1
TUBA1B
TUBA1C
KIF2C
TUBB4B
TUBG1
ACTC1
MMP1
TUBA1A
TUBB2A
COL3A1
MYO19
ROCK1
COL5A1
ACTA2
ACTG2
1.5
1
0.5
0
−0.5
−1
−1.5
B
Extracellular matrix organization
Extracellular structure organization
Skin development
Collagen metabolic process
Microtubule polymerization or depolymerization
Membrane docking
Cytoskeleton−dependent intracellular transport
Mesenchyme development
Microtubule polymerization
Ciliary basal body−plasma membrane docking
Organelle localization by membrane tethering
Mesenchyme morphogenesis
Collagen fibril organization
Extracellular matrix disassembly
Regulation of amyloid−beta clearance
Count
3
4
5
6
7
8
9
p.adjust
0.0005
0.0010
0.0015
0.10
0.15
0.20
0.25
GeneRatio
C
R2=0.9066
P<0.0001
Ace-tubulin/GAPDH
SOX9/GAPDH
R2=0.4305
P=0.0205
COL II/GAPDH
D
Cell viability (%)
N(nM) 0 5 10 25 50
D(nM) 0 0.5 1 2.5 5 10
E
SOX9
COL10A1
COL2A1
RUNX2
COL1A1
Reltive mRNA level
Relative mRNA level
ns
N (nM) - 5 10 25 - - -
D (nM) - - - - 0.5 1 2.5
F
Ctrl
D
E
Ace-tubulin
COL II
SOX9
GAPDH
52
142
70
37
(kDa)
Proteins/GAPDH
Ace-tubulin
SOX9
COL II

Fig.S6. Public data analysis of cytoskeletic genes and biological process in OA cartilage.

(A) Heatmap and **(B)** gene ontogeny analyses for cytoskeleton-associated genes in articular cartilage from healthy (Control) and osteoarthritis (OA) donors. **(C)** Relative analysis of the expression level of Ace-tubulin and SOX9, Ace-tubulin and COL II, respectively. **(D)** CCK8 analysis of the cell viability in chondrocytes (CHs) when treated with the nocodazole (N) and docetaxel (D) in different concentration, n=5. **(E)** RT-qPCR analyses of *SOX9, COL2A1, COL1A1, RUNX2,* and *COL10A1* in human CHs treated with nocodazole (N) and docetaxel (D) for 1 week. n=5. 2.5 nM of docetaxel had significant effects on chondrogenesis in CHs. **(F)** Western blot analysis of Ace-tubulin, SOX9 and COL II in CHs treated with docetaxel (D) and epothilone (E) for 1 week. Quantification of western blot data, n=5. Data are represented as the mean ± SD. $^{**}P<0.01$, $^{***}P<0.001$.

Fig. S7

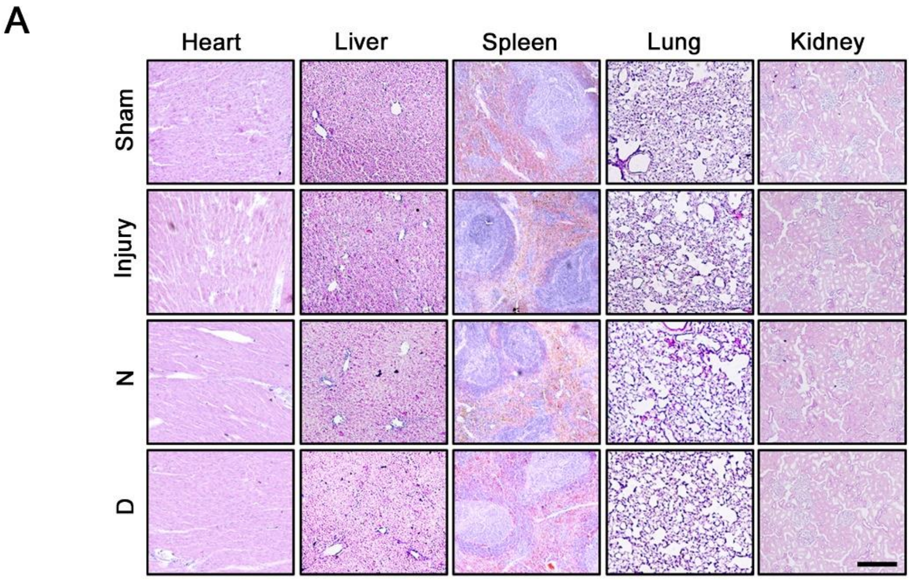

Fig S7. The effect of nocodazole (N) and docetaxel (D) *in vivo* and the safety *in vitro*

(C) H&E staining of various tissues other than cartilage from the rat cartilage injury model after 6-weeks treatment of N and D. There was no side effect in the tissues. Scale bar, 200 μm.

1 Fig.S8

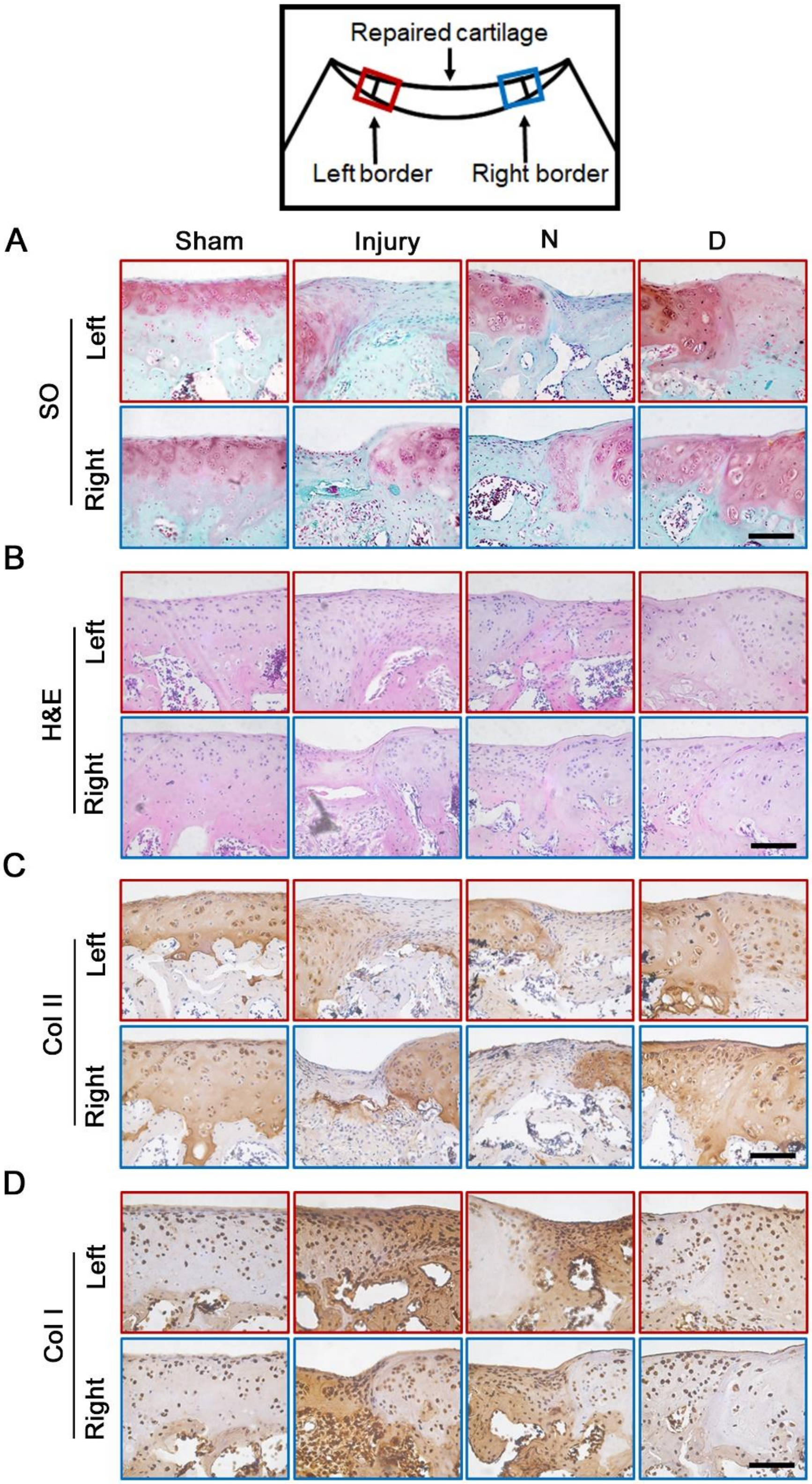
Repaired cartilage
Left border
Right border
A
Sham
Injury
N
D
SO
Left
Right
B
H&E
Left
Right
C
Col II
Left
Right
D
Col I
Left
Right

2

Fig S8. Histology of repaired cartilage of the rat cartilage injury model treated with nocodazole (N) and docetaxel (D).

(A) H&E staining, **(B)** Safranin O (SO) staining, and immunohistochemical staining for **(C)** type II collagen (Col II) and **(D)** type I collagen (Col I). The intra-articular injection of docetaxel improved cartilage repair in the model. The repaired cartilage was similar to the uninjured cartilage and its boundaries appeared blurred. Scale bar, 100 μm.

1 Fig.S9

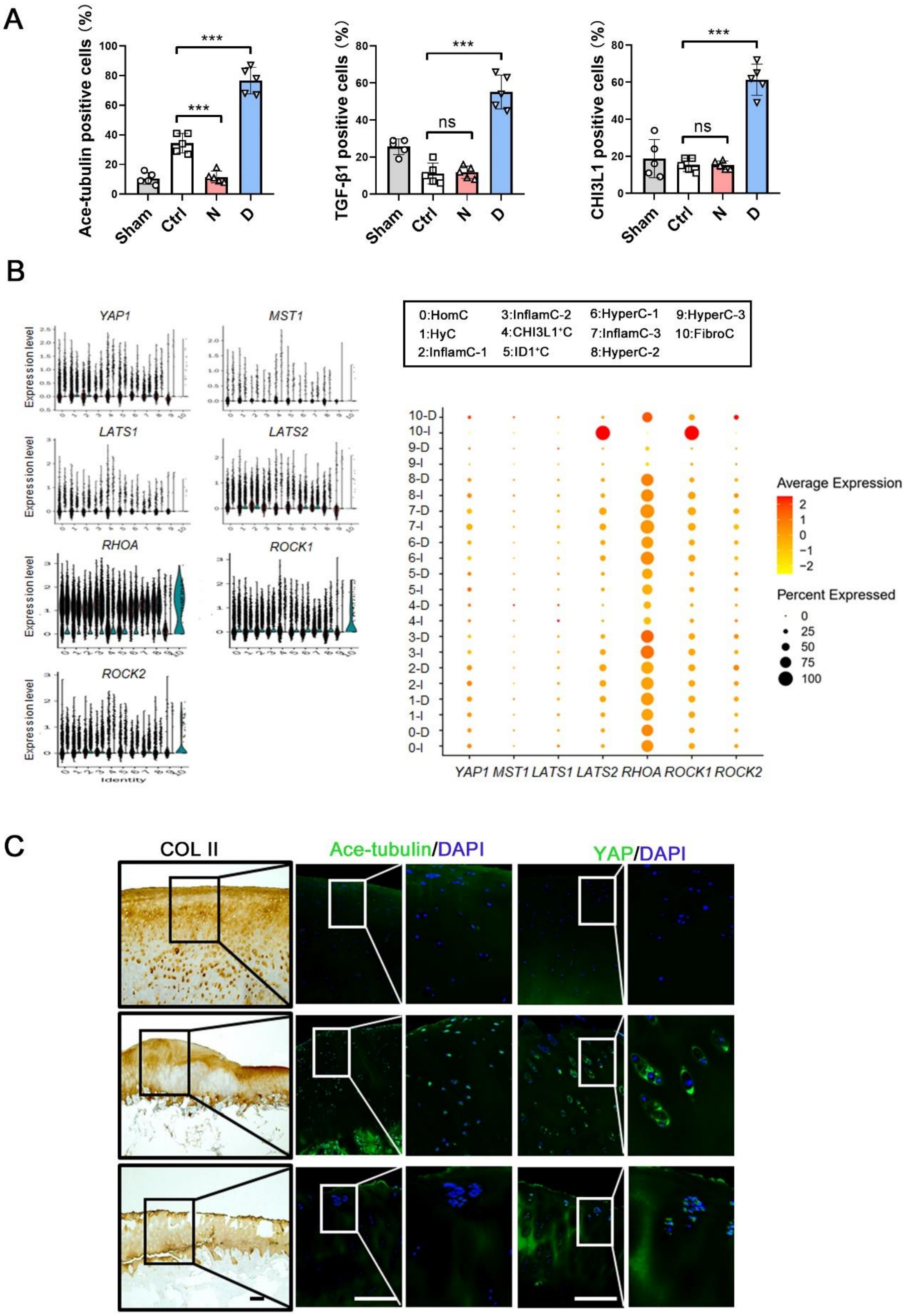
A
Ace-tubulin positive cells（%）
Sham
Ctrl
N
D
***
TGF-β1 positive cells（%）
ns
CHI3L1 positive cells（%）
B
YAP1
MST1
LATS1
LATS2
RHOA
ROCK1
ROCK2
Expression level
Identity
0:HomC
1:HyC
2:InflamC-1
3:InflamC-2
4:CHI3L1⁺C
5:ID1⁺C
6:HyperC-1
7:InflamC-3
8:HyperC-2
9:HyperC-3
10:FibroC
Average Expression
Percent Expressed
C
COL II
Ace-tubulin/DAPI
YAP/DAPI

2

Fig S9. The role of microtubule stabilization to YAP and YAP related signaling.

(A) Quantification of data Fig 4E (left), 4F (middle), and 4G (right).

(B) The violin plots and dot plots showing the expression of YAP and YAP related genes in each subpolpution between intact and damaged cartilage.

(C) Immunohistochemical staining for Col II, and immunofluorescence staining for Ace-tubulin and YAP in the cartilage depicting three different levels of abrasion severity. Scale bar, 200 μm.
